# SPRTN-dependent DPC degradation precedes repair of damaged DNA: a proof of concept revealed by the STAR assay

**DOI:** 10.1093/nar/gkad022

**Published:** 2023-01-31

**Authors:** Mateo Glumac, Mirjana Polović, Anja Batel, Andrea Gelemanović, Boris Maček, Ana Velić, Ivana Marinović-Terzić

**Affiliations:** University of Split School of Medicine, Laboratory for cancer research, Split 21000, Croatia; University of Split School of Medicine, Laboratory for cancer research, Split 21000, Croatia; University of Split School of Medicine, Laboratory for cancer research, Split 21000, Croatia; Mediterranean Institute for Life Sciences (MedILS), Split 21000, Croatia; Quantitative Proteomics, Interfaculty Institute of Cell Biology, Faculty of Science, University of Tuebingen, Tuebingen 72076, Germany; Quantitative Proteomics, Interfaculty Institute of Cell Biology, Faculty of Science, University of Tuebingen, Tuebingen 72076, Germany; University of Split School of Medicine, Laboratory for cancer research, Split 21000, Croatia

## Abstract

DNA-protein crosslinks (DPCs), formed by the covalent conjugation of proteins to DNA, are toxic lesions that interfere with DNA metabolic processing and transcription. The development of an accurate biochemical assay for DPC isolation is a priority for the mechanistic understanding of their repair. Here, we propose the STAR assay for the direct quantification of DPCs, sensitive to physiologically relevant treatment conditions. Implementing the STAR assay revealed the formation of small cross-linked peptides on DNA, created by the proteolytic degradation of DPCs by SPRTN. The initial proteolytic degradation of DPCs is required for the downstream activation of DNA repair, which is mediated through the phosphorylation of H2Ax. This leads to the accumulation of DNA repair factors on chromatin and the subsequent complete removal of the cross-linked peptides. These results confirmed that the repair of DPCs is a two-step process, starting with proteolytic resection by SPRTN, followed by the repair of the underlying damage to the DNA.

## Graphical Abstract

**Graphical Abstract:**
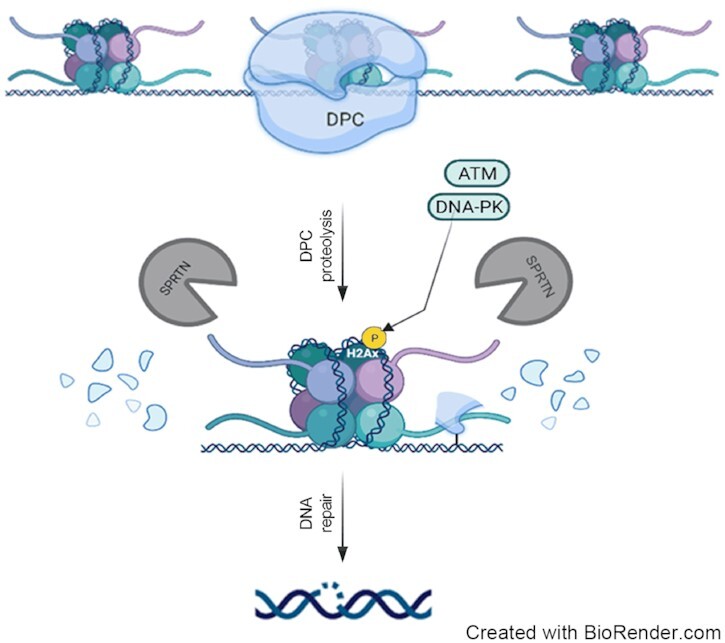
The first step in DPC repair is SPRTN-mediated proteolysis. The remaining cross-linked peptides are finally resolved by the activation of DNA damage repair.

## INTRODUCTION

DNA-protein crosslinks (DPCs) are toxic DNA lesions that originate from the covalent binding of proteins on DNA molecules following exposure to physical or chemical reagents, or as a result of faulty enzymatic reactions. Their toxicity stems from their interference with DNA metabolic processes, leading to genome instability, cancer, and aging (1). DPCs were first reported in 1962 when researchers observed a lower DNA recovery rate from UV-irradiated bacteria in a dose-dependent manner (2). They can be created by a myriad of cellular or environmental agents, such as reactive metabolic intermediary species (e.g. formaldehyde), ionizing radiation, ultraviolet light, heavy metals, and reactive chemical compounds. The fact that DPCs can be formed by exogenous agents has been successfully utilized for chemotherapy. DNA metabolic enzymes, which form transient intermediates with DNA, can become trapped on the DNA molecule, forming enzymatic DPC when exposed to specific chemical inhibitors. These include topoisomerase inhibitors, such as camptothecin (CPT), topotecan (TOP) and etoposide; DNA methyltransferase inhibitors, such as 5-aza-2′-deoxycytidine; or PARP1 inhibitors, such as olaparib. Other chemotherapeutic agents, such as platinum-based chemotherapeutics (cisplatin) or even radiation therapy, form non-enzymatic/unspecific DPCs as a part of their cytotoxic effect (3–7). DPCs have become a highly researched topic following the discovery of DNA-dependent metalloprotease wss1 in yeast (8) and its paralog SPRTN in mammalian cells (9,10) as the first dedicated enzymes in the DPC repair pathway. Since then, multiple proteases have been implicated in DPC repair (7,11,12). Because SPRTN is a promiscuous protease capable of degrading any DNA-bound protein, its activity is highly regulated. The regulation includes a ubiquitin switch (where deubiquitination activates its proteolytic activity), and acetylation and phosphorylation switches (which facilitate DNA binding) (13–18). DNA bases that have become crosslinked to proteins are damaged (chemically changed) and require repair to keep the DNA in good condition and to prevent functional problems. Canonical DNA repair pathways, such as homologous recombination (HR) (19–21), nucleotide excision repair (NER) (1,22,23) and Fanconi anaemia pathways (4,24), have been implicated in DPC repair. These pathways are usually mutually exclusive and can be activated by DPCs in different manners (5,11,21,22). How and why different DNA repair pathways are activated to resolve a specific DPC is not well understood. The initiation of DNA damage repair is conducted through ATR, ATM, and DNA-dependent protein kinases (DNA-PKs), which phosphorylate downstream targets, initiating the signalling cascade (25). Both ATM and DNA-PKs have been implicated in the repair of DNA damage induced by TOPO1 inhibitors, where a loss of function, either by gene silencing, knockout models, or chemical inhibition, leads to the accumulation of TOPO1-DPCs (14,26–30). Furthermore, it was shown that treatment with a TOPO1 inhibitor, leads to activation of ATM, which phosphorylates DNA-PK, which in turn phosphorylates H2Ax at the site of DNA damage, and that the disruption of the upstream signal diminishes its phosphorylation (29). The question remains as to how SPRTN and proteolysis are linked to the initiation of the repair of such lesions and overall DPC-induced DNA damage.

The successful detection of DPCs is a prerequisite for studying their formation, repair, and biological significance. Several methods have been developed for DPC detection. Depending on the design of the method, DPCs can be detected indirectly or directly (by marking the protein or by immunodetection). The indirect detection methods include alkaline elution, nitrocellulose filter binding, sodium dodecyl sulphate (SDS)/potassium chloride (KCl) precipitation, and single-cell gel electrophoresis. They are based on the separation of DNA fragments containing proteins from clean DNA fragments and report the results as a ratio of these quantities (5). A major advantage of indirect methods is that they do not require complete DNA purification for DPC detection. The downside is the nonlinearity between DNA and cross-linked proteins, which makes it difficult to quantitatively interpret the results obtained from these methods. Direct DPC detection methods include the ^125^I-post labelling and fluorescein isothiocyanate (FITC)-labelling methods. Both methods require highly purified DNA and protein labelling. The major advantage of direct DPC detection methods is the proportionality of the obtained signal and the amount of DPCs (5). The most commonly used immunodetection method is the RADAR assay. It requires DNA purification and the known identity of crosslinked proteins. The major advantage of this method is that it allows the study of specific DPC-associated proteins, therefore, it is commonly used to study enzymatically created DPCs. Disadvantages of this method include lower quantifiability than direct detection methods and a high dependence on sample purity (31).

Using different methods to study DPCs, we noticed specific problems, such as low sensitivity to DPC-inducing treatments with the requirement for high doses (sometimes higher than lethal doses), high background signals, incompatibility with different treatments, and low specificity of isolation with high levels of RNA contamination. Thus, we developed an assay that combines the strengths of all three types of DPC isolation methods. It should allow for easier quantification of total DPCs as well as for the preservation of DPC-forming proteins for downstream applications, such as western blotting (WB) or mass spectrometry (MS). The main objectives were to reduce the amount of background impurities originating from non-covalently bound proteins and RNA contamination (RNA-protein crosslinks). By exploiting the physical properties of DNA, we separated it from soluble proteins and RNA, and then used stringent conditions to remove all non-covalently bound proteins from DNA, leaving only DPCs as a protein source in the final isolate. The resulting **S**uperior method for **T**rue DN**A–**protein crosslinks **R**ecovery (STAR) assay is fast and simple, does not require specialized equipment or chemicals, and is applicable for multiple DPC-inducing and detection strategies. We used the STAR assay to evaluate DPC formation and repair. Improvement in the sensitivity and selectivity of the STAR assay, when compared to common methods, allowed for the observation of proteolytically created peptides crosslinked to DNA. By performing different experimental setups, we determined that DPC repair is a two-step process involving proteolytic degradation of DPC-forming protein by SPRTN in the first step and in the second step the removal of cross-linked peptides and damaged DNA bases by activation of DNA repair through the ATM and H2Ax axis. The STAR assay is an indispensable tool for studying the dynamics of DPC repair.

## MATERIALS AND METHODS

### Cell culture, treatments, and cell counting

HeLa and U2OS cells were obtained from the German Collection of Microorganisms and Cell Cultures (DSMZ) and cultured in Dulbecco's modified Eagle's medium (D6429, Sigma-Aldrich) with 10% fetal bovine serum (F0804, Sigma-Aldrich) and 100 U of penicillin/streptomycin (P0781, Sigma-Aldrich) (further referred to as a growth medium) in a humidified incubator at 37°C and 5% CO_2_ atmosphere. For cell passaging and division, Trypsin-EDTA was used (T4049, Sigma-Aldrich). For gene silencing (of SPRTN) Lipofectamine RNAiMAX transfection reagent (Invitrogen 13778-150) was used to transfect siRNA (siCTRL, 5′-AGG UAG UGU AAU CGC CUU G-3′, Eurofins; siSPRTN, 5′-GUC AGG AAG UUC UGG UUA AUA-3′, Microsynth). Reagent and siRNA were diluted in Opti-MEM (Gibco, 31985062), mixed, and then incubated for 15 min at room temperature. It was added dropwise on 40–60% confluent cells and incubated for 48 h. For DPC induction cells were treated with formaldehyde (FA), Topotecan (TOP), UV irradiation, and cisplatin. For FA treatments, formaldehyde (37% solution p.a., Merck) was diluted in PBS and added to the complete growth medium. Concentrations ranged from 100 to 1600 μM and the treatment duration is indicated for each experiment. For TOP treatment, Topotecan hydrochloride hydrate (T2705, Sigma-Aldrich) was dissolved in DMSO at 50 mM stock solution, and cells were treated with 3–60 μM concentration diluted in a complete growth medium for the indicated duration. For UV treatment, cells were subjected to UV irradiation using a UV crosslinker (CL-508.G Crosslinker, Uvitec) and the intensity was measured with a UV sensor (UVX digital Radiometer E29567, UVP). Administered doses ranged from 1 to 24 mJ/cm^2^. For cisplatin (CIS-Diammineplatinum (P4394, Sigma Aldrich)) treatment, cisplatin was dissolved in DMSO at 100 mM stock solution, and cells were treated with 50–200 μM for 15 min. For the cell recovery experiment, following induction of DPC either by FA, TOP or UV, the growth medium containing the treatment was removed (also done for UV treatment), cells were washed three times with PBS, and a fresh growth medium was added. Cells were then allowed to recover for an indicated time and then they were collected for further analysis. Cell counting was performed on an Olympus CHX41 microscope (Olympus Corporation) using a Neubauer chamber. Cell viability was assessed by trypan Blue staining (T8154, Sigma).

### DPC isolation

#### STAR assay

Cells were lysed in **buffer 1** [50 mM Tris–HCl (pH: 7.4), 1 mM EDTA, 150 mM NaCl, 1% Triton X-100, 0.5% deoxycholate (DOC), 0.1% sodium dodecyl sulphate (SDS), and protease inhibitors cocktail (Complete tablets Easy pack 04693116001, Roche)] for 15 min on ice. Lysed samples were centrifuged at 16 000 × *g* for 15 min. Pellet containing DNA and other insolubles was dissolved in **buffer 2** [6 M guanidinium–HCl, 10 mM Tris–HCl (pH: 6.5), 20 mM EDTA, 4% Triton X-100, 0.1% SDS and 1% Dithiothreitol (DDT)]. DNA was obtained by ethanol precipitation (75% ethanol cut-off value). DNA was pelleted at 16 000 × *g* for 10 min. Obtained pellets were washed with 75% ethanol and centrifugation was repeated. Supernatants were discarded and leftover ethanol was evaporated. Pellets were resuspended in **buffer 3** (16 mM NaOH) and then partially neutralized with an equal volume of 40 mM Tris–HCl (pH: 7.5). Samples were split for DNA and protein quantification.

#### RADAR assay

The RADAR assay was performed as described in the original paper (31). Briefly, cells were lysed using RADAR lysis buffer, DNA was precipitated by ethanol, and samples were cooled at −20°C. Pellets obtained after centrifugation were washed with 75% ethanol, air-dried, dissolved in 16 mM NaOH, and partially neutralized with an equal volume of 40 mM Tris–HCl (pH: 7.5). Samples were split for DNA and protein quantification. In the further text, the RADAR isolation method is referred to as ‘the common assay’ for DPC isolation.

### DNA isolation, cell fractionation, and total cell lysates (TCLs)

Referent DNAs of cellular isolates and fractions were obtained using a commercially available reagent (**TRIzol**, 15596026, Thermo Fisher Scientific) following manufacturers’ instruction or by ethanol precipitations from cellular fractions or TCLs. Precipitated nucleic acids were dissolved in 16 mM NaOH partially neutralized with an equal volume of 40 mM Tris-HCl (pH: 7.5). In the further text, the TRIzol DNA isolation method is referred to as ‘the commercial assay’ for DNA isolation. Cell fractionation to separate nuclear and cytoplasmic fractions was performed as described previously (32). Briefly, cells were resuspended in a hypotonic buffer containing 10 mM HEPES (pH: 7.9), 10 mM KCl, 100 mM EDTA, 100 mM EGTA, 1 mM DTT and protease inhibitors (Complete tablets Easy pack 04693116001, Roche). After 15 min on ice, Nonidet P-40 was added up to 0.6% (v/v) and cells were gently mixed by inversion. Nuclei were pelleted by centrifugation at 400 × *g* for 5 min at 4°C. The supernatant (cytoplasmic fraction) was collected and nuclei were lysed in **buffer 1**. For fractionation of the nucleus onto nucleoplasm and chromatin fractions, nuclear pellets were lysed in **buffer 1** for 2 min and chromatin was pelleted by centrifugation at 16 000 × *g* for 15 min. For protein analysis, the pellet was resuspended in **buffer 1** supplemented with 5 mM MgCl_2_ and Benzonase nuclease (E1014, Sigma) and incubated for 15 min at 37°C. The scheme of cell fractionation is given in Supplementary Figure S1 A. TCLs were prepared by lysing cells in buffer 1 supplemented with protease inhibitors, 5 mM MgCl_2_, and Benzonase nuclease (E1014, Sigma) and incubated for 15 min at 37°C.

### DNA quantification

DNA concentrations were determined using spectrophotometry (NanoDrop 1000, Thermo Scientific). DNAs' quality and quantity were visualized using agarose gel electrophoresis and ethidium bromide staining. Samples obtained by common assay were treated with RNase A (10109169001, Roche) for 15 min at 37°C. All samples for DNA quantification by spectrophotometry were treated with Proteinase K (7528, Carl Roth) at 55°C for 1–2 h. DNA was obtained by ethanol precipitation followed by centrifugation at 16 000 × *g* for 15 min. The pellet was washed with 75% ethanol, residual ethanol evaporated and DNA was dissolved in ultrapure water.

### Protein quantification

Proteins were quantified using BCA assay following the ‘Microplate procedure’ of the manufacturers’ instruction (33) with slight modifications. Briefly, samples were pre-treated with Benzonase nuclease (E1014, Sigma) to remove nucleic acids from samples as they could interfere with the assay. The resulting protein samples were loaded onto a 96-well microplate. To ensure equal loading, the volume of the protein sample was calculated according to the concentration of DNA (usually 0.25–2 μg). BCA Reagent A and B were mixed in a 50:1 ratio and 200 μl of this working reagent was added to each well containing protein samples. After 30 min at 37°C, absorptions were measured at 562 nm using a Microplate photometer (HiPo MPP-96, Biosan). Relative protein content was determined by normalization using the control samples as a reference point.

### SDS-PAGE and western blot analysis

All samples analysed by SDS-PAGE and Coomassie staining or western blot (WB) were pre-treated with Benzonase nuclease (E1014, Sigma) and 5 mM MgCl_2_ and incubated for 15 min at 37°C. Samples were mixed with 6× Laemmli buffer and heated up to 95°C for 5 min. DNA amount was determined by agarose gel electrophoresis and ethidium bromide staining and was used for loading control. Samples isolated by the same method were loaded on a gel according to an equal amount of DNA (in the range of 0.5–4 μg). Proteins were separated at 35 mA per gel for 75 min and then visualized by staining the gels with Coomassie Brilliant Blue (CBB) staining. Transfer of proteins onto 0.45 μm pore size nitrocellulose membrane (GVS) was carried out in a wet tank Mini Trans-Blot^®^ cell (Bio-Rad) at 200 mA for 90 min. Membranes were blocked using TBS–5%BSA–0.1%NaN_3_. Immunoblotting was performed using multiple antibodies, listed in Supplementary Data. Signal detection was performed using ChemiDoc (Universal Hood II; Bio-Rad Laboratories, Inc.) using enhanced chemiluminescence (ECL) substrate (Lumigen).

### Mass spectrometry

Mass spectrometry (MS) was performed on DPC isolates from untreated cells or treated with 400 μM FA for 15 min and the nuclear isolates from fractionated cells. SDS-PAGE was performed and in-gel digestion with Trypsin was conducted as described previously (34). Extracted peptides were desalted using C18 StageTips (35) and subjected to LC–MS/MS analysis. LC–MS/MS analyses were performed on an Easy-nLC 1200 UHPLC (Thermo Fisher Scientific) coupled to an QExactive HF Orbitrap mass spectrometer (Thermo Fisher Scientific) as described elsewhere (36). Peptides were eluted with a 60 min segmented gradient at a flow rate of 200 nl/min selecting the 20 most intensive peaks for fragmentation with HCD. The MS data was processed with MaxQuant software suite v.1.6.7.0 (37). The iBAQ was enabled. Database search was provided against human (96 817 entries) UniProt database using the Andromeda search engine (38). Data analysis was performed using R v4.0.0 (https://www.r-project.org/) (39). After pre-filtering, 1921 identified proteins remained for further analysis. Gene IDs were used for functional annotation based on PANTHER (http://www.pantherdb.org/panther/summaryStats.jsp) (40) protein class annotation dataset. Pie charts were used as a graphical representation of the results. In addition, Venn diagrams were created using the R package *eulerr* v6.1.1 (https://www.r-project.org/) (41) to show the difference in the composition of DPCs. The top 10 most cross-linked proteins by FA were determined by comparing iBAQ values from samples treated with FA with iBAQ values from non-treated samples. The presence of low-molecular-weight peptides was determined by cutting each SDS-PAGE gel lane into two sections that were analysed separately, approximately at 60 kDa (determined by protein marker), prior to MS analysis. The presence of protein-specific peptides from proteins with high molecular weight (larger than 90 kDa set as a cut-off value for analysis) was analysed in both gel sections. If such peptides were present in a gel section containing proteins with <60 kDa size, they were considered products of DPC proteolytic degradation. The mass spectrometry proteomics data have been deposited to the ProteomeXchange Consortium via the PRIDE (42) partner repository with the dataset identifier PXD032763.

### Immunofluorescence

Immunofluorescence was performed on HeLa cells grown in a complete growth medium on sterilized coverslips. Cells were either untreated or treated with 400 μM of FA for 15, 30 and 60 min. After treatment coverslips were washed with cold PBS. Pre-extraction was performed twice with cold CSK buffer (10 mM HEPES–KOH pH 7, 300 mM sucrose, 100 mM NaCl and 3 mM MgCl_2_) supplemented with 0.2% Triton X-100 for 10 min. Cells were washed 2 times with cold PBS followed by fixation in 2% paraformaldehyde in PBS for 20 min at room temperature. After 3 washes with PBS, permeabilization was performed with 0.15% Triton X-100 for 20 min. Coverslips were washed 3 times with cold PBS and then blocking was done in 3% BSA in PBS for 1 h. Coverslips were then incubated for 2 h with the SPRTN antibody (in-house) diluted in 3% BSA in PBS followed by washing 4 times with 0.05% Tween 20 in PBS. Secondary antibody (AlexaFluor 488 donkey anti-rabbit IgG (H + L), Invitrogen A21206) was diluted in 3% BSA in PBS, and coverslips were incubated for 1 h in the dark. After four washes with 0.05% Tween 20 in PBS, mounting medium (Mowiol) with DAPI (Sigma-Aldrich) was applied. Images were obtained using an Olympus CHX41 microscope (Olympus Corporation).

### Alkaline comet assay

For visualization of DPC-induced DNA damage, HeLa cells were treated with FA, trypsinized, and resuspended in PBS. Resuspended cells were mixed with 2% low melting point (LMP) agarose cooled below 40°C after melting, and layered on top of 1%-agarose-covered microscopic slides. After 10 min of polymerization on ice, in-gel cell lysis was performed with the lysis buffer (2.5 M NaCl, 100 mM EDTA, 10 mM Tris–HCl, 1% Triton X-100, and 10% DMSO) for 1 h at 4°C. After lysis, slides were washed with cold PBS. Because FA-induced DPCs interfere with the formation of Comet tails (43), slides were incubated overnight with Proteinase K (7528, Carl Roth) in PBS at 4°C (Supplementary Figure S5D). Next, slides were soaked in 300 mM NaOH with 1 mM EDTA for 10 min and then subjected to electrophoresis in the same buffer at 300 A (∼30V) for 20 min. Slides were neutralized with 50 mM Tris–HCl (pH: 7.5) for 10 min and then stained with ethidium bromide for 5 min. After destaining, Comets were visualized using the Olympus CHX41 microscope (Olympus Corporation). Obtained pictures were analysed using ImageJ (https://imagej.nih.gov/ij/) (43) and OpenComet software (https://cometbio.org/), as described previously (44) following MIRCA recommendations (45). Scoring was performed in Microsoft Excel which is an in-built function of OpenComet software. Comets with no visible nuclei (apoptotic cells) were not included in the calculation.

### Statistical analysis

Statistical analysis was performed using GraphPad Prism (version 9). Experiments containing three or more groups with one variable were analysed using one-way ANOVA. Post hoc analysis was performed using Tukey's multiple comparison test between any two groups. For multiple variable experiments, two-way ANOVA was performed. Post hoc analysis was performed using Dunnett's or Sidak's multiple comparison tests. The Comet assay statistical analysis was performed in GraphPad Prism using Brown-Forsythe's ANOVA test for skewed (not Gaussian) data. For multiple comparisons, Dunnett's T3 multiple comparisons test was performed. Only results with a calculated probability value (*P*) below 0.05 (*P* < 0.05) were considered significant in any performed analysis. Symbols for different test significance levels are assigned as follows: not significant (ns) for *P* > 0.05, * *P* < 0.05, ** *P* < 0.001, *** *P* < 0.0001 and **** *P* < 0.00001. All data for measured variables were expressed as mean ± SD. The sample size was *n* ≥ 3 containing biological and/or technical replicates.

## RESULTS

### The design and validation of the STAR assay

**Figure 1.**
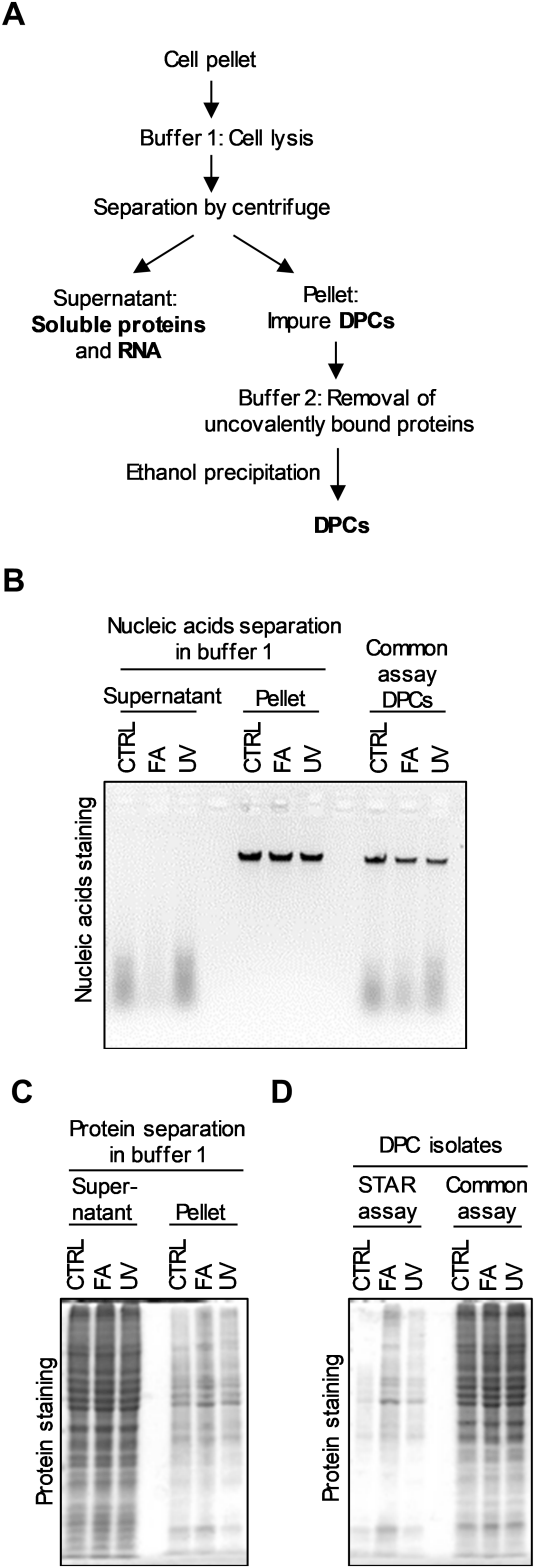
The design and validation of the STAR assay. (**A**) Schematic illustration of DPC isolation following the STAR assay protocols. (**B**) Separation of DNA/DPCs from the RNA in buffer 1 in the first step of the protocol. Cells were treated with FA (400 μM for 15 min) or UV (2 mJ/cm2, 5 min recovery), lysed in buffer 1, and centrifuged. Nucleic acids were visualized by agarose gel electrophoresis and ethidium bromide staining in parallel to the common assay isolates from the same cells. (**C**) SDS-PAGE analysis of isolates obtained from buffer 1. Proteins were visualized with CBB staining. (**D**) SDS-PAGE analysis of the same isolates pelleted from buffer 1 after cleansing with buffer 2 and ethanol precipitation, compared to the common assay isolates (both showing DPCs). Proteins were visualized with CBB staining.

We aimed to design a versatile assay for DPC isolation, which will be quick and simple to perform, with high DNA/DPC purity and minimal background protein signals, allowing for accurate protein quantification and identification, as well as for downstream analysis of DNA. The STAR assay is designed as a two-step process. In the first step DNA/DPCs are separated from the RNA and soluble proteins and in the second step isolated non-cross-linked proteins are removed from the DNA leaving only DPCs as a protein source in the sample (Figure 1A). We noticed that DNA could be efficiently extracted from cells lysed in buffer 1 by simple centrifugation, leaving RNA and almost all soluble proteins in the supernatant. Therefore, we tested whether this effect could be applied to reduce the RNA contamination and protein background signals observed in common methods for DPC isolation. The cells were treated with UV (2 mJ/cm^2^, 5 min recovery) or FA (400 μM for 15 min) to induce DPC formation. The isolation was performed side-by-side to common assay, for the comparison of DNA and protein yield. After lysis in buffer 1 (the first step in the STAR assay protocol), supernatants, and pellets were analysed on an agarose gel to stain nucleic acids with ethidium bromide, and SDS-PAGE was used to stain for proteins with CBB (Figure 1 B and C). DNA was completely pelleted, leaving almost all RNA in the supernatant (Figure 1B) while isolates from the common assay contained a high amount of RNA contamination. The protein pattern and content after first step of the STAR assay were similar in all three isolates with no difference between treatments both in the supernatants and pellets (Figure 1C). Buffer 2 was used to remove any non-covalently bound proteins from the DNA, leaving only DPCs as a protein signal in the sample. Buffer 2 contained high ion and detergent concentrations which created stringent conditions for the removal of protein impurities from DPC isolates. Pellets obtained after centrifugation from buffer 1 were treated with buffer 2 and DNA/DPCs were obtained by ethanol precipitation. SDS-PAGE and CBB showed an increasing amount of proteins in the UV- and FA-treated isolates compared to the control isolates, proving the higher presence of DPCs in the treated cells. DPCs obtained by the common assay contained a higher amount of proteins with no visible increase in treated samples (Figure 1D). To demonstrate the importance of removing RNA from the DPC isolates, supernatants obtained from untreated and FA-treated HeLa cells were subjected to ethanol precipitation. The pellet obtained was dissolved in buffer 2 to remove all non-cross-linked proteins from the RNA. The obtained RNA and proteins were analysed on an agarose gel, followed by ethidium bromide staining, and on SDS-PAGE, followed by CBB. The results showed that RNA could pollute DPC samples with a high amount of RNA protein crosslinks (Supplementary Figure S1B), reducing the noise-to-signal ratio which leads to lower sensitivity.

### The STAR assay is selective and sensitive assay for DPCs detection

**Figure 2.**
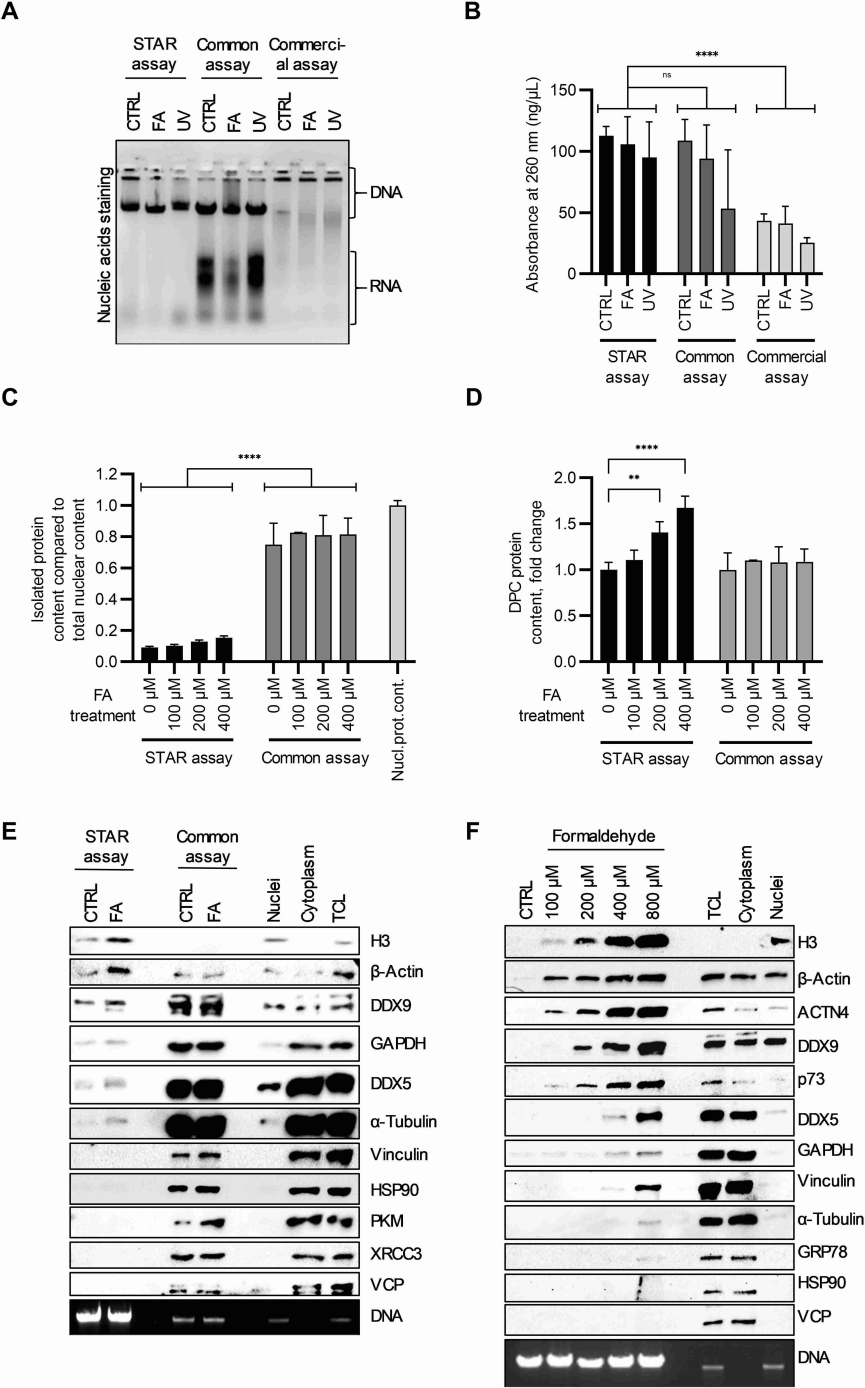
Specificity and sensitivity of the STAR assay towards DPCs. (**A**) Evaluation of specificity and efficiency of DNA recovery between different isolation protocols. DNA/DPCs were isolated following three comparable methods: the STAR assay, common assay, and commercial assay for DNA isolation from cells treated with formaldehyde (FA) (400 μM for 15 min) or UV (2 mJ/cm^2^, 5 min recovery). Isolates were evaluated by agarose gel electrophoresis and ethidium bromide staining. (**B**) After RNA digestion by RNAse DNA concentration was measured as absorbance at 260nm using spectrophotometry. The results were analysed by Dunnett's multiple comparisons test (main column effect). (**C**) Cells were exposed to 15-min FA treatment in indicated concentrations to induce crosslinks and DPCs were isolated using the STAR and the common assays. For referent control, the nuclear fraction was isolated and protein content was evaluated under the same conditions. The same amount of cells was used for each experiment. Protein content was evaluated by BCA assay. Presented statistical analysis is a result of one-way ANOVA. (**D**) Evaluation of assay sensitivity to FA treatment after normalization. Presented statistical analysis is Dennett's multiple comparisons test. (**E**) Immunoblot detection of selected endogenous proteins in DPCs isolated by two assays, with nuclear and cytoplasmic fractions and the total cell lysate (TCL) used as positive controls. DNA was used as a loading control. (**F**) Evaluation of the STAR assay sensitivity for DPC induction by immunoblot. DPCs were induced by 15-min FA treatment in indicated concentrations. Selectivity for nuclear localization was evaluated by comparing DPC isolates with nuclear fractions. DNA was used as a loading control. Symbols for different test significance levels are assigned as follows: not significant (ns) for *P* > 0.05, * *P* < 0.05, ** *P* < 0.001, *** *P* < 0.0001 and **** *P* < 0.00001. All data for measured variables were expressed as mean ± SD. The sample size was n = 3 containing technical replicates.

To evaluate the efficiency of DNA recovery by the STAR assay, we compared it to DNA recovered by the common assay for DPC isolation and by a commercially available reagent for DNA extraction (commercial assay). For this purpose, cells were either untreated or treated with FA (400 μM for 15 min) or UV (2 mJ/cm^2^, 5 min recovery). The cells were trypsinized and divided equally to ensure the same amount of starting material for isolation. Thus, we expected to obtain an equal amount of DNA from all isolation protocols. Isolation was performed using the STAR assay, the common assay, and the commercial assay. Agarose gel electrophoresis showed a similar efficiency of DNA recovery by both the common and STAR assays, but significantly less with a commercial assay (Figures 2A and B). Notably, the common assay isolates contained a high amount of RNA impurities, unlike the STAR assay and a commercially available assay. To improve DNA purity, needed as a loading control for downstream experiments, RNase A and proteinase K digestions were performed. After DNA precipitation, RNA removal was demonstrated on agarose gel with representative samples (Supplementary Figure S1C). We noticed that all three methods used for isolation extracted lower amounts of DNA following DPC-inducing treatments, but the reduction was small and not statistically significant compared to the corresponding controls. Next, we performed a specificity test for DPCs. The cells were treated with increasing concentrations of FA for 15 min to induce DPC formation. The cells were equally divided to create technical replicates for experiments. DPC isolation was performed using STAR and common assays, while an equal number of untreated cells was used for the isolation of nuclear proteins as referent control. The relative protein content was determined using the BCA assay (Figure 2C). The protein content in the DPC isolates obtained by the common assay was much higher than the protein content isolated by the STAR assay. Isolates from the common assay contained almost as much proteins as were extracted from control cells nuclei. These results demonstrated the lower specificity of the common assay with regard to uncross-linked nuclear proteins. When the obtained results were normalized to control samples for each assay group, the content of STAR assay-isolated DPCs significantly increased in dependence on FA concentration, which was not observed for the common assay isolates (Figure 2D). These results confirmed the higher sensitivity and specificity of the STAR assay for DPCs. To test whether the obtained results could be extrapolated to other cell lines, a DPC isolation experiment was performed on FA-treated or untreated U2OS cells. DPCs were isolated using the STAR and common assays. The results obtained for the two cell lines complement each other. The amount of DNA extracted was equivalent for both methods. RNA impurities were present in the common assay isolates from U2OS cells, as confirmed by RNase A digestion. Protein content was much higher in the common assay isolates, but no increase in DPC protein content was observed after FA treatment, whereas an increase was observed in the STAR assay isolates (Supplementary Figures S1D and E). To test the specificity towards individual DPC-forming proteins, DPCs were isolated by both the STAR and the common assays, and western blot analysis was performed. DPCs isolated from untreated cells (CTRL) and cells treated with 400 μM FA for 15 min were compared to the nuclear and cytoplasmic fractions and TCL of untreated cells. DNA was used as a loading control. Western blot results (Figure 2 E) show that the majority of tested proteins were present in the common assay isolates without an observable increase after FA treatment. The STAR assay isolates confirmed an increase in the protein amount after FA treatment, which supports the results obtained by BCA. The STAR assay isolates provided several negative results in both the untreated control and FA-treated samples, which supports its specificity towards DPCs. Comparing this result with results from cell fractionation showed that proteins that did not blot in the STAR assay isolates were not present in the nuclear fraction, further confirming the assay's specificity towards DPCs. Also, histones, which create the majority of endogenously formed DPC (46) were among the most sensitive proteins to DPC induction isolated by the STAR assay, but not by the common assay. To validate the sensitivity of the STAR assay to DPC induction by low doses and short treatments of FA, DPCs were induced with increasing concentrations of FA for 15 min and then isolated using the STAR assay. Western blot results showed a correlation between the applied FA dose and the amount of immunodetected proteins in the isolates, which confirmed high sensitivity of the STAR assays. Proteins with high nuclear abundance were easily crosslinked to DNA, even at low FA concentration (100 μM in the short treatment). Proteins with lower nuclear abundance required higher FA concentrations to crosslink to DNA, whereas proteins without nuclear localization could not crosslink to DNA at all tested FA concentrations (Figure 2F). These results suggest that nuclear localization is a prerequisite for protein cross-linking to DNA molecules, validating the high specificity of the STAR assay for DPCs. Further, we tested whether DPCs generated by other DPC-inducing agents, such as topotecan (TOP), ultraviolet irradiation (UV), and cisplatin could be detected by the STAR assay. Although these agents induce other types of DNA damage in addition to DPCs, we demonstrated that the STAR assay efficiently isolates TOP-, UV-, and cisplatin-induced DPCs (Supplementary Figure S2A–D).

### The MS analysis of DPC forming proteins identifies the presence of proteolytically created DNA-peptide crosslinks

**Figure 3.**
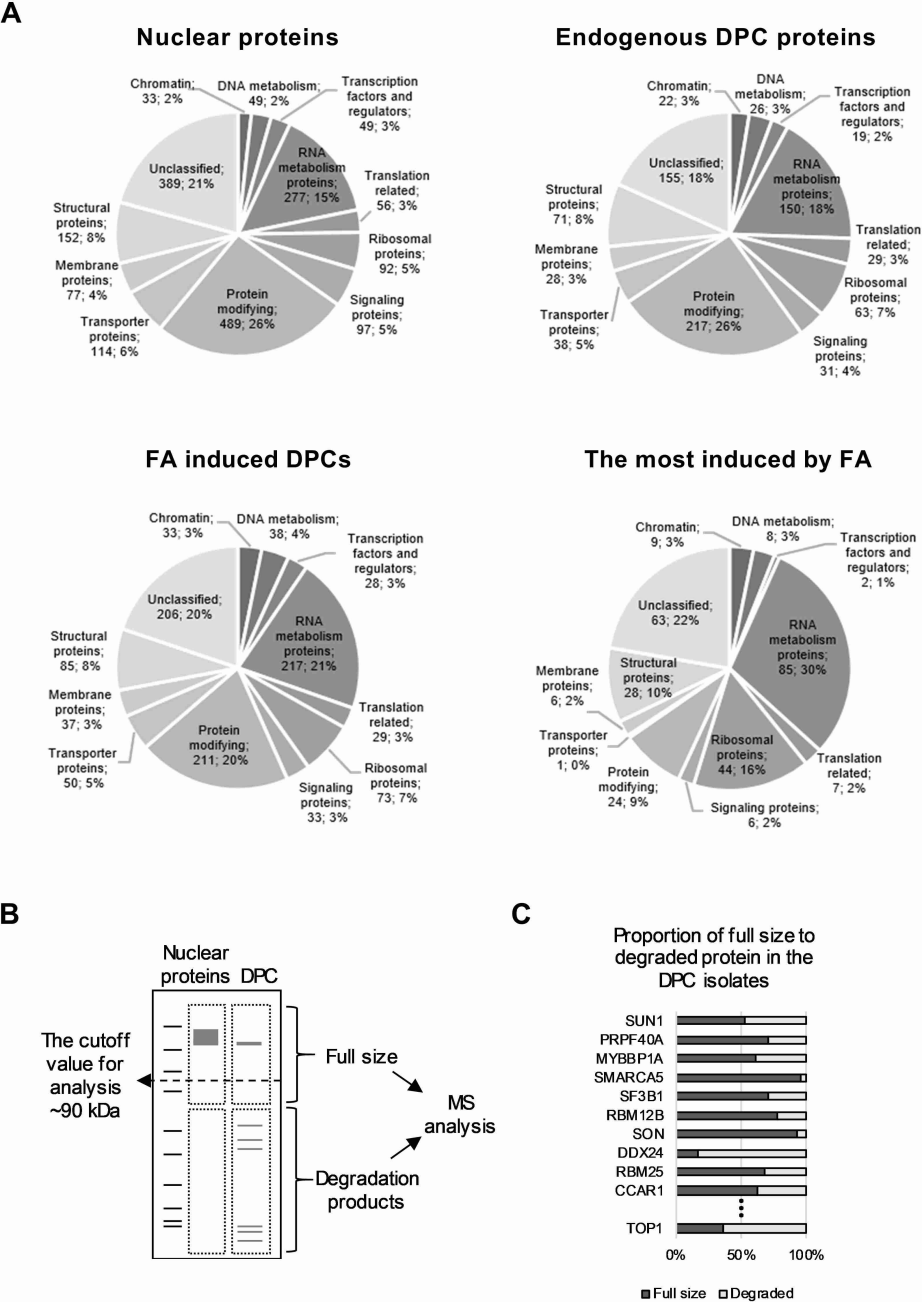
The MS analysis of DPC-forming proteins identified the presence of proteolytically created DNA-peptide crosslinks. (**A**) MS analysis performed on DPC isolates from untreated cells (Endogenous DPC-proteins) or cells treated with 400 μM FA for 15 minutes (FA-induced DPCs) and from nuclear isolates (Nuclear proteins). Identified proteins were categorized into 11 larger categories according to functional annotation based on the PANTHER dataset class annotation. The data are presented as pie charts representing the proteins identified in the nuclear fraction, endogenous DPCs, FA-induced DPCs, and the most induced by FA. (**B**) Schematic representation of MS analysis designed to detect the presence of proteolytically formed DNA-peptide crosslinks. DPC isolates from untreated and FA-treated cells and nuclear fractions were subjected to SDS-PAGE prior to MS analysis. After separation, lanes containing isolates were cut out of the gel and further cut at 60 kDa size, determined by the protein marker. Two gel sections were analysed separately. (**C**) The proportion of full-size proteins larger than 90 kDa (detected in the gel section containing >60 kDa proteins) in the total amount of identified specific peptides corresponding to a specific protein. The proteins presented are the top 10 most induced nuclear proteins by FA treatment (determined by iBAQ values) and one of the most studied DPCs, the TOPO1.

To analyse the composition of DPCs and to further test the isolation specificity, we performed mass spectrometry (MS) analysis on DPC isolates from untreated HeLa cells or cells treated with 400 μM FA for 15 min and from nuclear fraction isolates. Samples were separated according to size by SDS-PAGE, and each sample line was cut out of the gel and divided into two fragments with a cut-off size of 60 kDa. Each gel fragment was analysed separately. After pre-filtering, 1921 identified proteins remained for further analysis. To present the composition of DPCs in relation to nuclear localization, Venn diagrams were created, showing overlaps between the DPCs, nucleoplasm, and chromatin fraction. To show endogenous DPC composition, samples ‘Nucleoplasm’, ‘Chromatin’ and ‘Endogenous DPCs’ were used, and to show FA-induced DPC composition, samples ‘Nucleoplasm’, ‘Chromatin’ and ‘FA induced DPCs’ were used. The obtained results confirmed that nuclear localization is a predisposition for protein crosslinking to DNA molecule, as there was only a 2–6% difference between proteins contained in DPC isolates and proteins found in the nuclear fractions (Supplementary Figure S3). The analysis was qualitative, and proteins were included without regard for their abundance in the sample. Next, the PANTHER protein class annotation dataset was used and proteins were categorized into 11 larger categories: ‘Chromatin’, ‘DNA metabolism’, ‘Transcription factors and regulators’, ‘RNA metabolism proteins’, ‘Translation related’, ‘Ribosomal proteins’, ‘Signalling proteins’, ‘Protein modifying’, ‘Transporter proteins’, ‘Membrane proteins’ and ‘Structural proteins’. Pie charts were created to show the qualitative composition of the proteins annotated to categories, presenting: i) nuclear proteins, from protein detected in both nucleoplasm and chromatin samples, ii) endogenous DPCs and iii) ‘FA induced DPCs’ (Figure 3A). A comparison of pie charts for nuclear proteins and endogenous DPC proteins showed a great similarity. From the FA-induced DPC protein subgroup, it can be observed that the protein categories involved in DNA and RNA metabolic processes are over-represented in the DPC composition. To determine which protein categories showed the greatest level (percent) of change in response to FA treatment, the subgroup ‘The most induced by FA’ was calculated for each category as the first quartile by iBAQ values from ‘FA induced DPCs’ data. The results indicate that ‘Ribosomal proteins’ and ‘RNA metabolism proteins’ are protein categories the most induced by formaldehyde treatment. By analysing each sample in two separate sections (Supplementary Figure S4A), we tested for the presence of DPC degradation peptides still cross-linked to DNA. Proteins with molecular masses above 90 kDa were analysed to avoid possible cross-contamination with the lower gel fragments (Figure 3B). Figure 3C shows the 10 most highly induced nuclear proteins by treatment with FA (also shown in Supplementary Figure S4B) and, in addition, the highly explored TOPO1-DPCs, as the proportion of full-size to degraded protein found in the endogenous DPC isolates. The presence of peptides specific for proteins larger then 90 kDa in the lower gel section (containing proteins below 60 kDa) was detected in the DPC isolates, whereas their presence was almost undetectable in the nuclear fractions. This confirms the presence of degradation peptides in DPC isolates. We also show that FA induces an increase in the amount of peptides after only 15 min of treatment (Supplementary Figure S4C).

### The presence of cross-linked peptides on the DNA molecule correlates with DPC proteolysis

**Figure 4.**
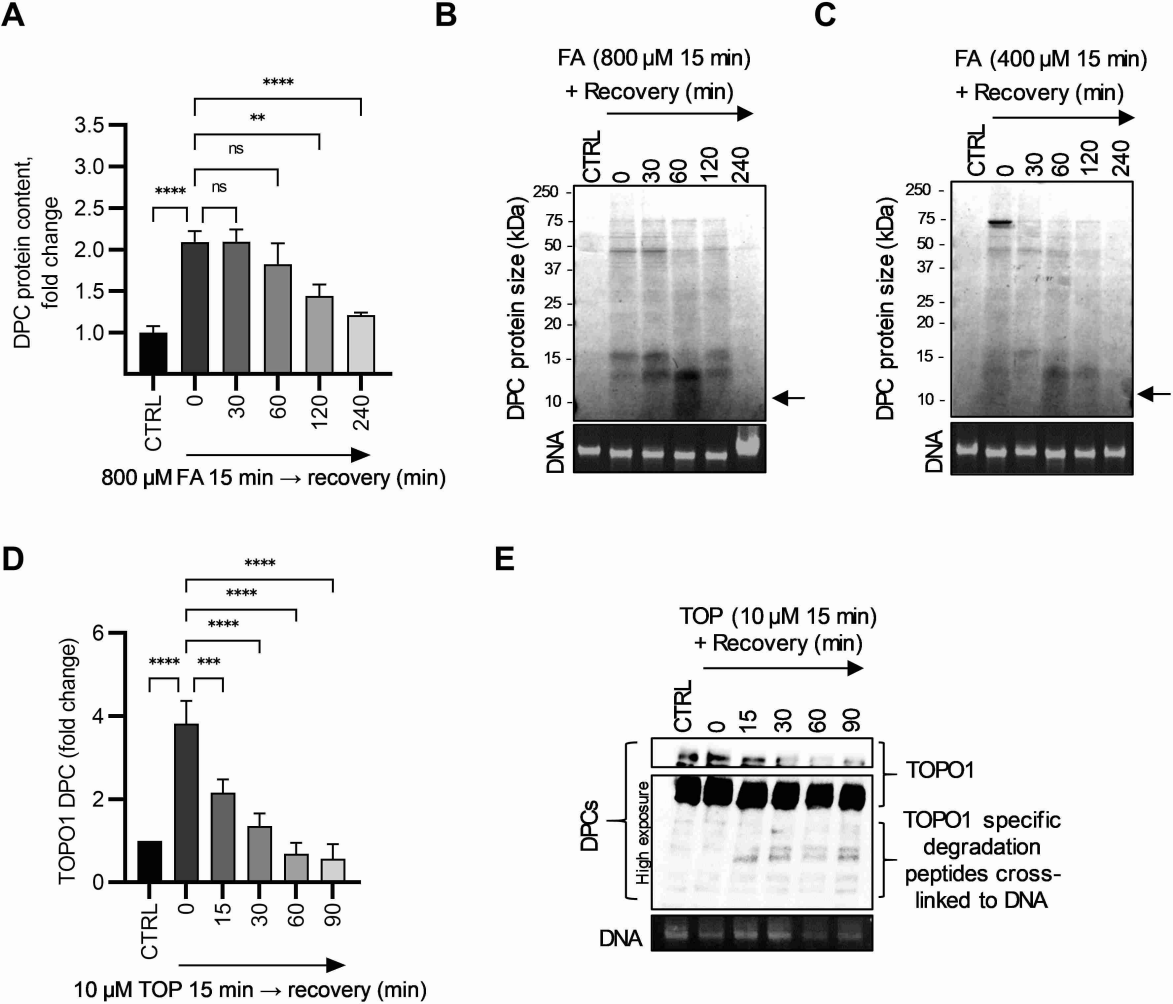
The presence of cross-linked peptides on the DNA molecule correlates with DPC proteolysis. (**A**) The dynamics of resolving DPCs was evaluated in a recovery experiment. Cells were treated with 800 μM FA for 15 minutes and allowed to recover for indicated time duration. DPCs were isolated by STAR assay and quantified using BCA assay. The presented statistical analysis is the result of Tukey's multiple comparisons test. (**B**) Detection of proteolytically created peptides still crosslinked to DNA in DPC isolates from recovery experiment. Cells were treated with 800 μM FA for 15 min and allowed to recover for indicated time duration. DPCs were isolated by STAR assay and SDS-PAGE CBB staining was performed. (**C**) The same experimental setup as B but DPCs were induced by 400 μM of FA for 15 min. (**D**) The dynamics of DPC repair after Topotecan (TOP) induction of TOPO1-DPCs. Cells were treated with 10 μM of TOP for 15 min and left to recover for an indicated time. DPCs were isolated by STAR assay and quantified by immunodetection using a TOPO1 antibody. The presented statistical analysis is the result of Dunnett's multiple comparisons test. (**E**) Western blot detection of proteolytically created Topoisomerase peptides still crosslinked to DNA during recovery experiment after TOP treatment. Symbols for different test significance levels are assigned as follows: not significant (ns) for *P* > 0.05, * *P* < 0.05, ** *P* < 0.001, *** *P* < 0.0001 and **** *P* < 0.00001. All data for measured variables were expressed as mean ± SD. The sample size was *n* = 3 containing biological replicates.

To demonstrate the dynamics of repair, cells were treated with 800 μM FA for 15 min to induce DPCs and then were left to recover to allow for DPC repair. DPCs were isolated using the STAR assay and quantified using the BCA assay. The results show DPC induction after treatment with FA. As the recovery time increased, the protein content in the DPC isolates decreased demonstrating the efficient DPC repair (Figure 4A). After 4 h of recovery time, the protein content in DPCs isolates decreased almost to the baseline level. The decrease in protein content in DPC isolates was also observed in CBB staining for 800 and 400 μM FA (Figure 4B and C). The decrease in protein content was accompanied by an accumulation of cross-linked proteolytically degraded peptides (indicated by an arrow), with a peak occurring at 60 min recovery time. Removal of these cross-linked peptides occurred with a longer recovery time. We demonstrated the same effect with TOP. After treatment with 10 μM TOP for 15 min, cells were left to recover. DPCs were isolated using the STAR assay and TOPO1-DPCs were detected using WB. The results show a much faster repair rate than after treatment with FA, with TOPO1-DPC levels returning to baseline level in less than 60 min (Figure 4D). Similar to the treatment of FA, degradation products were also observed for TOPO1 (Figure 4E), with the reduction starting after 60 min of recovery. The removal of the degradation peptides from DNA is significantly delayed behind the onset of the proteolytic degradation of the full-size protein.

### SPRTN chromatin binding proceeds the activation of DNA damage repair

**Figure 5.**
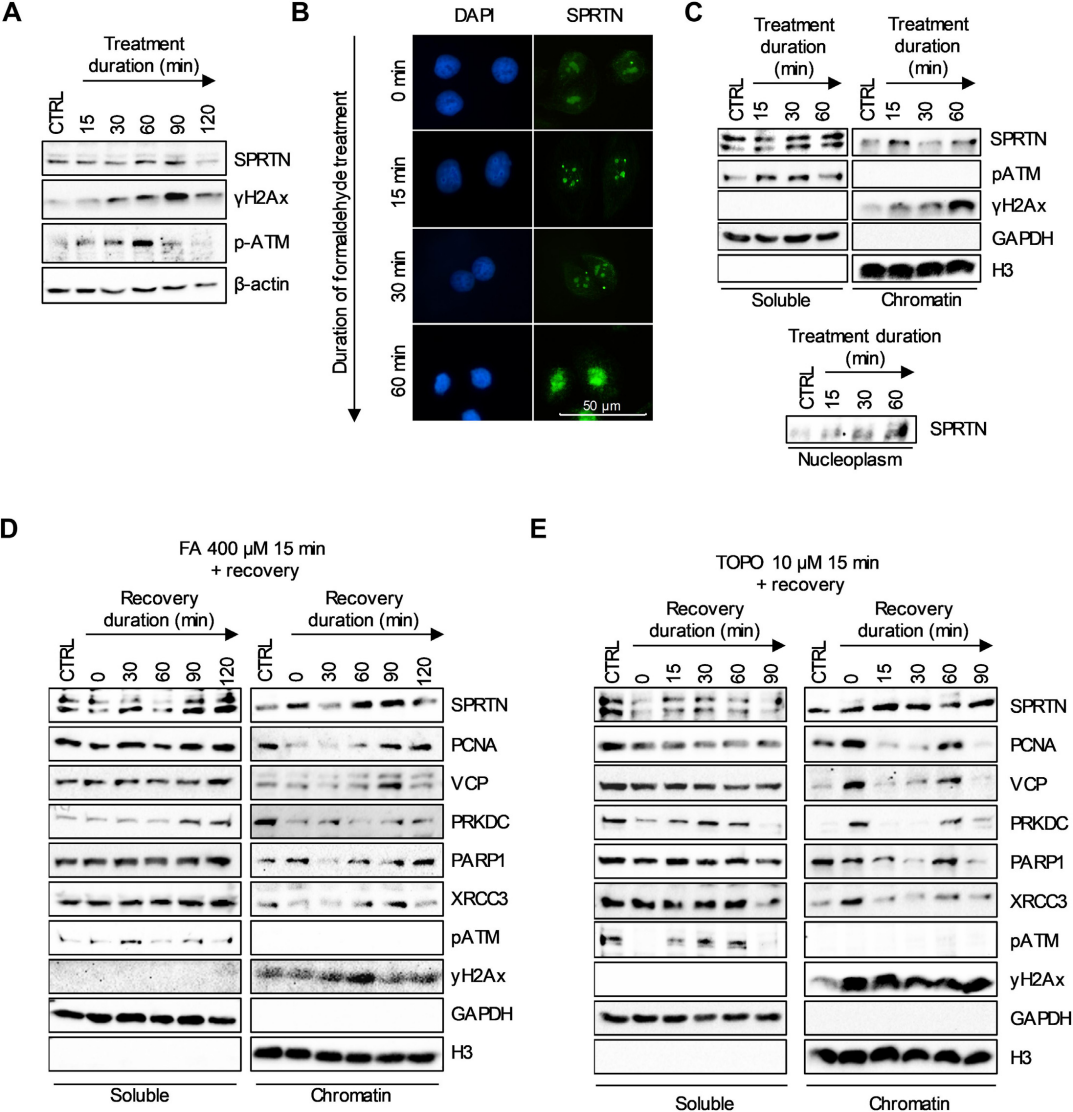
SPRTN chromatin binding in relation to DNA damage signalling and the loading of DNA repair factors. (**A**) DNA damage signalling and SPRTN expression were analysed in cells treated with 400 μM of FA for the indicated time using WB. Actin was used as a loading control. (**B**) Immunofluorescence imaging of SPRTN following the treatment with 400 μM of FA for the indicated time. (**C**) Cell fractionation of cells treated with 400 μM FA for indicated time presented as soluble versus chromatin fraction. GAPDH and H3 were used as loading controls for each fraction. Additionally, the nucleoplasmic fraction was blotted to show a nuclear accumulation of SPRTN. The nucleoplasmic fraction was loaded equivalently to soluble and chromatin fractions. (**D**) Cell fractionation of FA-treated cells (400 μM for 15 min) recovered for indicated time presented as soluble versus chromatin fraction. GAPDH and H3 were used as loading controls for each fraction. The chromatin binding of SPRTN, PCNA, VCP, PRKDC, PARP1, and XRCC3 was evaluated in accordance with DNA damage signalling (p-ATM and γH2Ax). (**E**) Cell fractionation of TOP-treated cells (10 μM for 15 min) recovered for indicated time presented as soluble versus chromatin fraction. GAPDH and H3 were used as loading controls for each fraction. The chromatin binding of SPRTN, PCNA, VCP, PRKDC, PARP1 and XRCC3 was evaluated in accordance with DNA damage signalling (p-ATM and γH2Ax).

To identify the underlying DNA repair signals involved in DPC repair, we performed WB analysis of TCLs from FA-treated HeLa cells (400 μM). The results indicate an increase in SPRTN expression in treated cells, with a peak around 60 min. ATM phosphorylation correlated with SPRTN expression, which also peaked around 60 min of treatment. γH2Ax activation lagged behind SPRTN expression and phosphorylation of ATM (Figure 5A). Using the same treatment, SPRTN was analysed with IF. SPRTN formed foci following FA treatment and then in the 60 min time-point there is a striking increase in SPRTNs nuclear presence (Figure 5 B). To demonstrate how much of SPRTN detected in the nucleus was bound to chromatin, cell fractionation was performed. WB analysis revealed that the increase in SPRTN chromatin binding after treatment with FA had two peaks: after 15 min of treatment, and again at 60 min (Figure 5C). Blotting of the nucleoplasmic fraction obtained in the same experiment showed that increasing amounts of SPRTN were present in the nucleus, peaking at 60 min of treatment, consistent with our IF results. Increasing SPRTN chromatin binding and presence in the nucleoplasm was followed by an increase in ATM phosphorylation and activation of γH2Ax after 60 min. Performing the same experiment on cells treated with TOP, we found immediate activation of γH2Ax and continuously increasing SPRTN chromatin binding (Supplementary Figure S5A). Because a continuous treatment constantly generates DPCs that could interfere with the repair process, we performed all further experiments during the recovery period after a short treatment. HeLa cells were treated with FA (400 μM) or TOP (10 μM) for 15 min and then allowed to recover for an indicated time. Cells were fractionated and analysed to detect the presence of SPRTN and DNA repair factors on chromatin. After treatment with FA, the soluble (non-chromatin) amount of SPRTN decreased immediately after treatment, whereas SPRTN chromatin binding again showed two peaks, increasing immediately after 15 min of treatment (recovery time 0 min) and again after 60 and 90 min of recovery time. As before, p-ATM followed SPRTN and peaked before γH2Ax. After γH2Ax, DNA repair factors increasingly loaded onto chromatin including PCNA, VCP, PRKDC, PARP1, and XRCC3 (Figure 5D). The loading of DNA repair factors correlates well with the onset of removal of degradation peptides from DNA, as we have shown in the previous figure. For the TOP treatment and recovery experiment, cell fractionation revealed a high presence of DNA repair factors at the beginning of the recovery experiment, followed by their immediate detachment from DNA at a later time point. The detachment of DNA repair factors was not accompanied by the detachment of SPRTN from chromatin. The presence of SPRTN on chromatin increased, as did the phosphorylation of ATM and γH2Ax. The high SPRTN presence on chromatin correlated with the time points of highest TOPO1-DPC degradation, as shown in the previous figure. Similar to FA treatment, the soluble amount of SPRTN decreased immediately after TOP treatment. The results for both FA and TOP treatments indicate that SPRTN is produced by cells in response to the formation of DPCs, which increases the requirement for SPRTN activity. DNA repair factors were reloaded onto DNA after 60 min of recovery, the time point at which removal of TOPO1-specific degradation peptides began (Figure 5E). SPRTN chromatin binding was followed by p-ATM, which in turn was followed by γH2Ax, resulting in the loading of DNA repair factors onto chromatin.

### Proteolytic degradation of DPCs, by SPRTN, is essential for signalling and the repair of DPC-induced DNA damage

**Figure 6.**
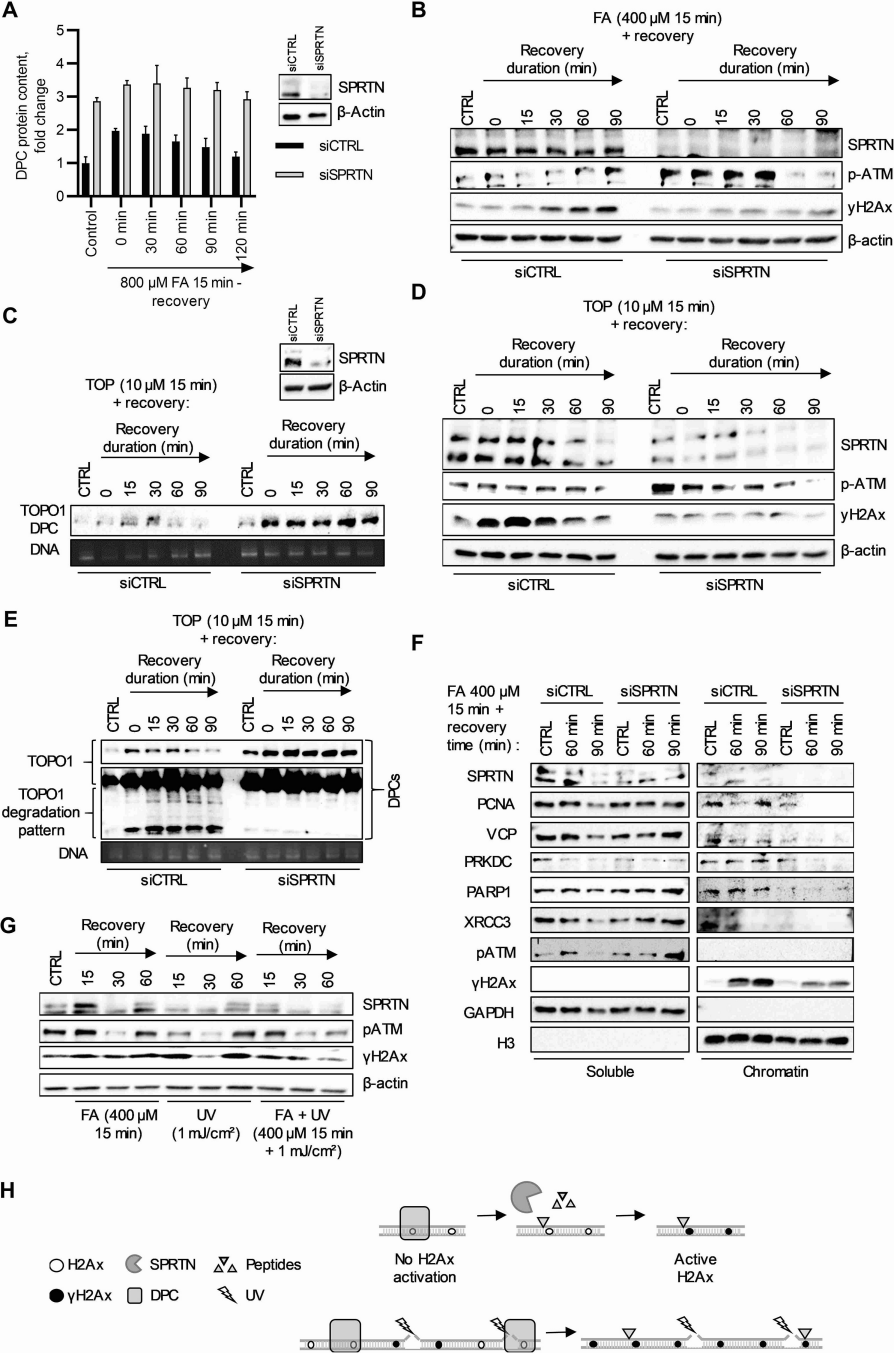
Proteolytic degradation of DPCs, by SPRTN, is essential for signalling and the repair of DPC-induced DNA damage. (**A**) The dynamics of resolving DPCs in siCTRL and siSPRTN silenced cells were evaluated in a recovery experiment. The efficiency of SPRTN silencing was demonstrated by WB. Cells were treated with 800 μM FA for 15 min and allowed to recover for indicated time duration. DPCs were isolated by STAR assay and quantified by BCA assay. (**B**) The association of DNA damage signalling and SPRTN was analysed by WB in siCTRL and siSPRTN silenced cells treated with 400 μM of FA for 15 min and left to recover for the indicated time. Actin was used as a loading control. (**C**) The dynamics of resolving TOP-induced DPCs in siCTRL and siSPRTN silenced cells were evaluated in a recovery experiment. The efficiency of SPRTN silencing was demonstrated by WB. Cells were treated with 10 μM TOP for 15 minutes and allowed to recover for indicated time duration. DPCs were isolated by STAR assay and quantified by immunoblotting using the DNA as a loading control. (**D**) The association of DNA damage signalling and SPRTN was analysed by WB in siSPRTN silenced cells treated with 10 μM of TOP for the indicated time duration. Actin was used as a loading control. (**E**) Dependency of DPC proteolysis on SPRTN. Cells were treated with 10 μM TOP for 15 minutes and allowed to recover for indicated time duration. DPCs were isolated by STAR assay and quantified by immunoblotting using the DNA as a loading control. (**F**) Cell fractionation of siCTRL and siSPRTN cells treated with 400 μM of FA for 15 min and left to recover for 60 and 90 min. GAPDH and H3 were used as loading controls for each fraction. The chromatin binding of SPRTN, PCNA, VCP, PRKDC, PARP1, and XRCC3 was evaluated in accordance with DNA damage signalling (p-ATM and γH2Ax). (**G**) The dependence of pATM and γH2Ax activation on the presence of DPCs. Cells were either treated with 400 μM of FA for 15 min, UV (1mJ/cm^2^), or the combination of FA treatment followed by UV treatment, and left to recover for 15, 30 and 60 min. Actin was used as a loading control. (**H**) DPCs pose a steric obstacle to the activation of γH2Ax. DPC degradation, by SPRTN, is critical for the activation of γH2Ax in the vicinity of DPCs.

To confirm the importance of SPRTN for proteolytic degradation of DPCs, SPRTN expression was reduced using siRNA in HeLa cells. Cells were transfected with either siCTRL or siSPRTN and incubated for 48 h. After incubation, cells were trypsinized and divided into multiple plates for the experiment. DPCs were induced by treatment with either FA (800 μM) or TOP (10μM) for 15 min and allowed to recover for the indicated time before collection. DPCs were isolated using the STAR assay, and SPRTN silencing efficiency was confirmed using WB. Cells transfected with siCTRL were efficient in repairing FA-generated DPCs (similar to Figure 4 A), whereas DPC repair was impaired in siSPRTN cells (Figure 6A and Supplementary Table S1). To investigate the effects of SPRTN silencing on p-ATM and γH2Ax, transfected cells were lysed and WB analysis was performed. The results show that successful SPRTN silencing also leads to strong phosphorylation of ATM in the control cells. γH2Ax was activated after the high DPC degradation in siCTRL cells, but this effect was absent in cells transfected with siSPRTN (Figure 6B). The same experimental setup using TOP to induce DPCs yielded the same results. siSPRTN cells were inefficient at removing TOPO1-DPCs from DNA (Figure 6C). In addition, H2Ax was not phosphorylated in siSPRTN cells (Figure 6D). DPCs isolated from cells with siCTRL showed an accumulation of crosslinked degradation peptides, starting from the beginning up to 60 min of the recovery time, after which their quantity started to decrease (Figure 6E). Cells transfected with siSPRTN showed no proteolytic degradation of TOPO1-DPCs and no reduction in overall TOPO1-DPC amount. The time point in which the removal of DNA-peptides started correlated with the binding of DNA repair factors shown in Figure 5E. Because we have previously shown that activation of γH2Ax is followed by loading of DNA repair factors, we performed cell fractionation of siCTRL and siSPRTN cells treated with 400 μM FA for 15 min and recovered for 60 and 90 min to encompass the time-points with expected activation of γH2Ax activation and loading of DNA repair factors. The results showed efficient activation of γH2Ax in siCTRL cells followed by loading of DNA repair factors at the subsequent time point, whereas in siSPRTN cells, activation of γH2Ax was impaired, and DNA repair factors were not loaded onto chromatin (Figure 6F). This demonstrates the importance of γH2Ax activation for DNA repair factor loading. To determine if SPRTN silencing will affect γH2Ax activation in a DPC-independent manner, we treated siCTRL and siSPRTN cells with hydrogen peroxide (H_2_O_2_, 100 nM) and UV (1 mJ/cm^2^). We found that treatment with DNA-damaging agents, which predominately induce non-DPC DNA damage, resulted in efficient γH2Ax activation, despite silencing of SPRTN (Supplementary Figure S5B and C). This confirms that SPRTN is involved in γH2Ax activation in a DPC-dependent manner. To determine whether SPRTN itself or its proteolytic removal of DPCs was the cause of γH2Ax activation, cells were pre-treated cells with 400 μM of FA for 15 min, to induce DPCs followed by induction of DNA damage by UV (1 mJ/cm^2^). The cells were allowed to recover for 15, 30 and 60 min. The idea behind this experiment was to create protein barriers in the form of DPCs, that would interfere with the repair of DNA damage other than DPC. Treatment with FA alone replicated previous results, showing an increase in γH2Ax after 60 min of recovery. UV alone strongly activated γH2Ax. Pre-treatment with FA, followed by UV treatment, resulted in decreased activation of γH2Ax compared with UV treatment alone (Figure 6G). These results suggest that the removal of the protein barrier by degradation of DPCs, rather than by SPRTN alone, leads to the activation of γH2Ax at sites of DNA damage (Figure 6H).

### The effect of DPC repair on cell proliferation and DNA integrity

**Figure 7.**
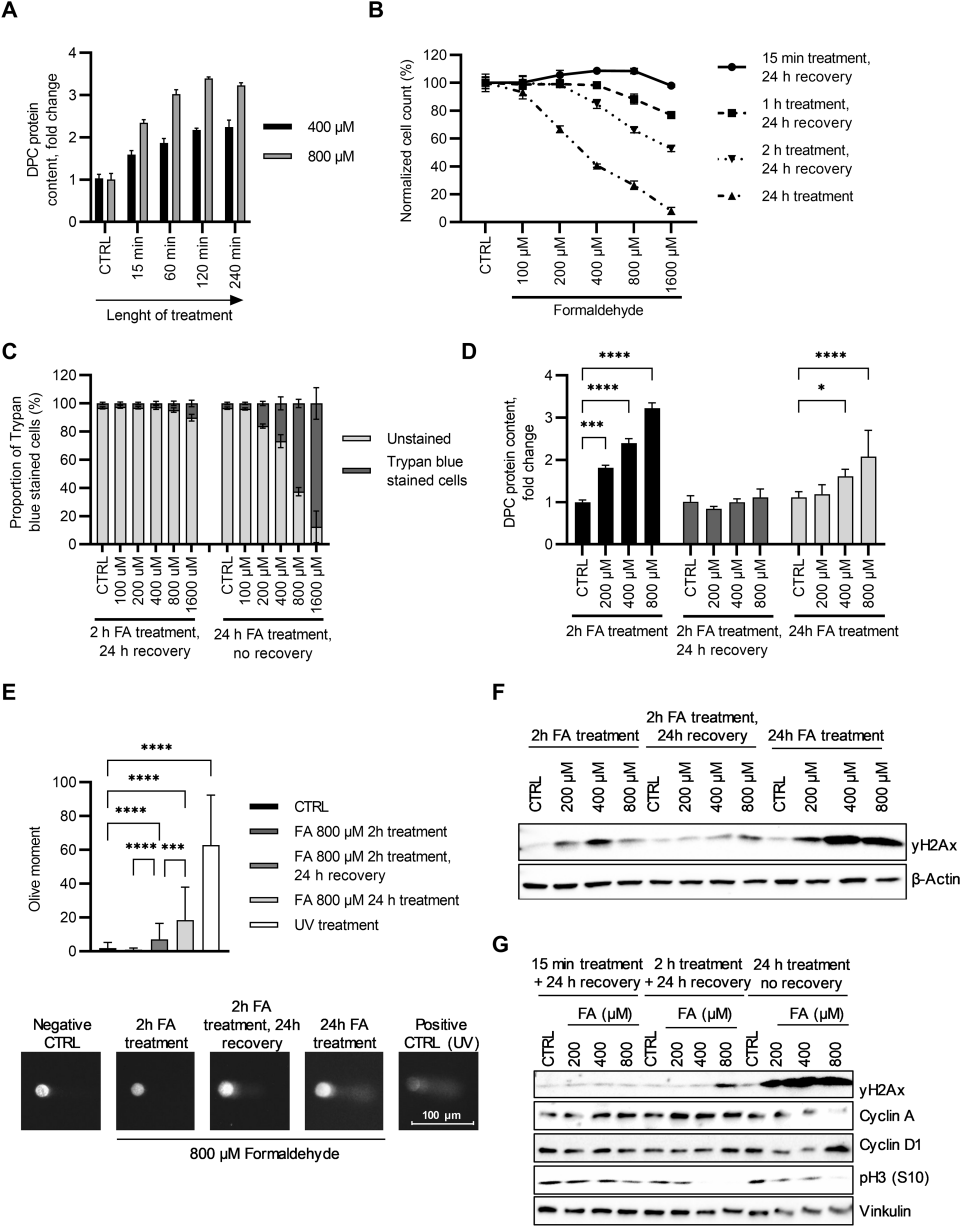
DPC repair leads to lower proliferation and DNA breaks. (**A**) DPC content obtained from cells treated with 400 and 800 μM FA for indicated treatment duration. DPCs were isolated by the STAR assay, quantified using the BCA assay, and normalized to untreated control. (**B**) The impact of FA treatment in a time and concentration-dependent manner was evaluated by cell counting. Cells were treated with FA for indicated concentration and length of time, counted by microscopy, and normalized to untreated controls. (**C**) Cell viability was assessed by trypan blue staining. Data presented are normalized cell counts to the total number of cells for the particular treatment conditions. (**D**) Repeating the same experimental setup as in (B), with 2 h treatment as a positive control for DPC induction, the efficiency of DPC resolving was evaluated. DPCs were isolated using the STAR assay and the results were normalized to untreated control. Statistics presented are results of Dunnett's multiple comparisons test. (**E**) The same experimental setup was used in order to evaluate the induction of DNA breaks by FA treatment. Using the harshest treatment (800 μM FA), an alkaline comet assay was performed and the results presented belong to three biological replicates with at least 100 cells included in the evaluation (* in 24 h treatment 68 apoptotic cells were excluded from the quantification). Representative comets are shown below. The statistic presented are results of Dunnett's T3 multiple comparisons test. (**F**) Immunoblot probing for γH2Ax in TCLs from the same experimental setup. Actin was used as a loading control. (**G**) Immunoblot of γH2Ax and cell cycle markers: cyclin A and D1, and phosphorylated form of histone H3 (S10); in cells treated with different concentrations of FA with or without recovery. Actin was used as a loading control. Symbols for different test significance levels are assigned as follows: not significant (ns) for *P* > 0.05, * *P* < 0.05, ** *P* < 0.001, *** *P* < 0.0001 and **** *P* < 0.00001. All data for measured variables were expressed as mean ± SD. The sample size was *n* = 3 containing biological replicates.

To determine how the duration of treatment affects the amount of DPCs formed by treatment with FA, cells were treated with 400 and 800 μM FA in combination with increasing treatment duration. DPCs were isolated using the STAR assay and quantified using the BCA assay. The results show a strong accumulation of DPCs up to 120 min after the start of treatment, after which no further accumulation of DPCs was observed for either treatment concentration (Figure 7A). As expected, 800 μM produced significantly more DPCs than 400 μM (two-way ANOVA, *P* < 0.0001). Since both treatment duration and concentration affected the accumulation of DPCs, we tested how different amounts of DPCs generated by the treatment with FA would affect cell proliferation and viability. Hela cells were treated with different concentrations of FA in combination with different treatment duration and then allowed to rest for 24 h. As shown in Figure 7 B, both the treatment duration and the concentration of FA affected the reduction in cell count. Cells treated with FA for 15 min showed no decrease in cell count regardless of the concentration applied. Cells treated for longer periods (1 and 2 h) showed a significant decrease in cell count, compared to 15 min treatment followed by 24h recovery period (two-way ANOVA, *P* < 0.0001), with the effect being more pronounced at higher FA concentrations. Continuous FA treatment for 24 h had the strongest impact on cell count and the effect was stronger with higher concentrations of FA. To distinguish whether the decrease in cell count was due to a decrease in cell proliferation or an increase in cell death, we performed a trypan blue staining assay. The results showed that cells recovered after treatments with higher concentrations of FA proliferated more slowly and did not die, whereas cells treated continuously for 24 h showed a sharp increase in trypan blue staining, indicating increased cell death (Figure 7 C). Cells treated with FA and allowed to recover for 24 h did not die after treatment, indicating efficient DPC repair. To compare the dynamics of DPC induction and repair after treatment with different FA concentrations, we repeated the recovery experiment. Cells were treated for 2 h and then collected or allowed to recover for 24 h. Continuous treatment for 24 h was used as a positive control. DPCs were isolated, and the results are shown in Figure 7D. Cells treated for 2 h without recovery showed a significant increase in the amount of DPC in correlation with the concentration of FA (as shown previously). Cells that were left to recover for 24 h had a DPC amount reduced to baseline level, whereas cells treated continuously for 24 h had more DPCs than baseline levels but less than cells treated for 2 h, indicating efficient DPC repair in both experimental setups. Because the protein part of DPCs had been successfully removed, we wanted to find the reason for the lower cell proliferation and cell death in our experiments. To test whether treatment with FA induces DNA breaks, cells were treated with 800 μM FA in the same treatment pattern as in the previous experiment. An alkaline comet assay was performed to quantify the amount of DNA breaks. The results shown in Figure 7 E demonstrate that after 2 h of treatment cells had no DNA damage detected by the comet assay, proving that the FA treatment alone does not induce DNA breaks. In contrast, cells treated for 2 h and recovered for 24 h showed increased DNA breaks compared with control or 2 h treated cells. Cells treated for 24 h exhibited an even higher level of DNA breaks, as expected according to cell proliferation and viability assays. These results indicated that DNA breaks form following DPC proteolysis and are the culprits for reduced cell count observed in a cell proliferation assay. Blotting γH2Ax showed an increase in γH2Ax level in a 2-hour treatment period, indicating high DNA repair at this time point. Phosphorylation continued to increase after 24 h of continuous treatment, indicating a high amount of DNA damage not limited to DPCs. Curiously, cells treated for 2 h and then left to recover had less γH2Ax than cells in 2 h treatment without recovery, suggesting that repair was less active, although the comet assay showed an increase in DNA damage. This result implies the distinction between γH2Ax function in DPC repair and the repair of DNA damage that occurs after the removal of the protein part of DPCs (Figure 7F). For further validation corresponding to DPC repair and inhibition of cell proliferation, cells were treated with 200, 400 and 800 μM of FA for 15 min or 2 h and then recovered for 24 h and compared with cells that were continuously treated for 24 h. Immunoblotting revealed concentration and time-dependent γH2Ax activation. Cell cycle markers were used to determine in which phase of cell cycle cells accumulated following these three experimental setups (Figure 7G). Cyclin A was used as an S and G2/M marker (48). Cyclin D1 was used as a G1 phase marker (49). Histone H3 is phosphorylated at serine 10 in cell mitosis (50). Results showed an increase of cyclin A in both recovery experimental setups. In the continuous treatment, cyclin A was reduced corresponding to a high proportion of cell death. Cyclin D1 increased with 800 μM FA both in 2 h treatment + 24 h recovery and particularly in 24 h continuous treatment. Phospho-H3 (S10) decreased in all treatments and correlated with treatment dosage and duration. Taking all cell cycle markers into consideration cells were accumulating in the S phase of the cell cycle in recovery experiments, indicating that leftover peptides cause the lengthening of the S phase.

## DISCUSSION

In this study, we described the development of the STAR assay, an assay for two-step DPC isolation, that can be performed in any laboratory with little or no investments. The STAR assay is a selective, sensitive, and versatile method for direct quantification of DPCs capable of detecting non-enzymatic and enzymatic DPCs using multiple DPC-inducing agents (FA, UV, TOPO, and cisplatin) and DPC detection strategies such as gel staining, BCA assay, immunoblotting, and mass spectrometry. The STAR assay reduces background protein signal ∼8-fold compared with the commonly used RADAR assay, which is the most cited assay for DPC isolation, developed for direct DPC quantification (4,10,14,17,18,51). We have found that RNA contamination and the associated RNA-protein crosslinks create a high protein background that conceals DPCs when direct DPC quantification is performed. Indirect methods such as the ARK assay (4) and the K-SDS method (52), which primarily quantify cross-linked DNA rather than proteins, are less affected by RNA contamination. In addition, the STAR assay was validated with SPRTN knock-down models, in which we found that a reduction in SPRTN expression leads to an accumulation of endogenous DPCs as well as an impairment of DPC repair after induction by FA or TOP.

The STAR assay proved to be highly successful in detecting peptide residues left over from proteolysis of DPCs using MS, protein staining, and WB. Their existence was previously demonstrated in two different experimental approaches: using proteins cross-linked to plasmid DNA (1,53) and using a method for fluorescent labelling of proteins (21,22) on genomic DNA. The STAR assay showed a time-dependent accumulation of the cross-linked peptides on DNA that correlated with the formation of SPRTN nuclear foci and binding to chromatin, as shown by IF and cell fractionation. SPRTN chromatin binding precedes ATM phosphorylation. We demonstrated impaired DPC repair and accumulation of endogenous DPCs in SPRTN-silenced cells. Such cells exhibit high activation of ATM, which could be explained by a high amount of stalled replication forks, leading to activation of ATM, as shown by Halder et al (17). We have shown that downstream of p-ATM and SPRTN chromatin localization is the activation of H2Ax. γH2Ax is usually considered a marker for double-stranded DNA breaks (28,54–60), but it has been shown to be involved in other types of DNA repair pathways. γH2Ax could be activated by several pathways, including ATM and PRKDC (25,28,29,55,61,62). Following the onset of DPC proteolysis, ATM transmits the DNA damage signal to H2Ax. We also found that PRKDC is loaded onto chromatin after TOP and, to a very small extent, after FA. TOP generates TOPO1-DPC and a single-stranded DNA break at each lesion site. Greater involvement of PRKDC in the repair of such lesions is expected, as it has been shown to be involved in the repair of DNA breaks (62). Looking at the events after DPC induction, it appears that the repair of both FA- and TOP-induced DPCs follows the same mechanism: SPRTN present in the nucleus is immediately loaded onto chromatin to activate repair signalling. Being spent in that process (auto-proteolysis (16)), more SPRTN is produced, imported into the nucleus, and reloaded onto chromatin to initiate DPC proteolysis. Activation of H2Ax directs the loading of DNA repair factors such as PCNA, VCP, XRCC3 and PARP1. The activation and loading of DNA repair factors correlate with the initiation of proteolysis of cross-linked peptides on DNA, implying that the DNA repair mechanism is responsible for the complete removal of DPC. This sequence of events led us to conclude that DPC repair is a two-step process involving proteolytic degradation of the protein part of a DPC by the SPRTN protease, which leaves peptide residues still cross-linked to DNA, followed by the activation of DNA repair leading to its complete removal from DNA.

We also investigated the importance of DPC proteolysis for the subsequent activation of DNA repair. In cells with reduced expression of SPRTN, both FA and TOP induced DPC are not being proteolytically processed. This confirmed previous findings (4,10,30,6) and indicate that SPRTN is the major protease involved in proteolytic degradation of DPC. In addition, we found that silencing of SPRTN prevents γH2Ax activation in response to DPC-inducing agents (FA and TOP) but not to other DNA damage-inducing agents (such as UV and H_2_O_2_). Because γH2Ax activation is associated with DNA repair activation and DNA repair factor loading (28,54–60) this intrigued our interest. As a result of FA treatment, SPRTN-silenced cells accumulated DNA breaks (47), not because of direct DNA damage by FA, but because of DPC-induced replication fork stalling and transcriptional stress (10). Our results, together with previously published discoveries (10,47), demonstrate the importance of the proteolytic activity of SPRTN for the downstream repair of DNA lesions at the sites of DPCs. We have shown that XRCC3 is loaded onto DNA after SPRTN DPC proteolysis and γH2Ax activation. This implies that even proteolytically degraded peptides cross-linked to DNA would lead to the formation of DNA breaks, as found in FA-treated siSPRTN cells (47). This proves that DNA repair after DPC proteolysis is critical for genome stability. By inducing DPCs by FA and then damaging DNA by UV irradiation, we found that DPCs alone inhibit the activation of DNA repair, implying that DPCs present a steric barrier on DNA. The reduction of their size by proteolysis is required for the activation of DNA repair. Another observation we made is that DNA breaks occur even if the protein part of DPCs has been proteolytically degraded and can no longer be detected. This could be explained by the irregular distribution of H2Ax on chromatin. H2Ax is present in approximately 10% of nucleosomes (63), yet its distribution is not random but exists in clusters distributed on the chromatin (64). If the location of DPCs doesn’t contain H2Ax in its vicinity, the underlying DPC-DNA damage would not be processed until it is exposed to replication or transcription. We hypothesize that the main reason for the formation of the observed DNA breaks and the activation of γH2Ax 24h after DPC induction is the response to remaining DNA-peptide crosslinks. The aforementioned adducts would interfere with replication forks, causing DNA breaks (47) and subsequent activation of γH2Ax. This would also explain the slower cell proliferation and accumulation in the S phase, although DPCs were efficiently repaired in the same experimental setup. This suggests that not only DPCs but also underlying DNA damage could affect DNA metabolic processes (replication and transcription), leading to genomic instability observed in Ruijs-Aalfs syndrome (9). In conclusion, the improvement of DPC isolation and detection by the STAR assay revealed the dynamics of DPC repair. This allowed further mechanistic studies in which we demonstrated an important role of SPRTN in activating the DNA damage response in the presence of DPCs. The STAR assay proved to be an important tool for the study of DPCs.

## DATA AVAILABILITY

Software used in this paper:

- ImageJ, Schneider et al., 2012, https://imagej.nih.gov/ij/
- GraphPad Prism (version 9), GraphPad, https://www.graphpad.com/scientific-software/prism/
- OpenComet software, OpenComet, https://cometbio.org/
- R v4.0.0, R Foundation for Statistical Computing, https://www.r-project.org/
- PANTHER Classification System, http://www.pantherdb.org/panther/summaryStats.jsp
- MaxQuant software suite v.1.6.7.0, Max Planck Institute of Biochemistry, https://www.maxquant.org/

Results of MS analysis were deposited in the PRIDE depository database operated as a part of ProteomeXchange Consortium. Dataset identifier number PXD032763.

## Supplementary Material

gkad022_Supplemental_Files

## References

[1] Chesner L.N. , Campbell C. A quantitative PCR-based assay reveals that nucleotide excision repair plays a predominant role in the removal of DNA-protein crosslinks from plasmids transfected into mammalian cells. DNA Repair (Amst.). 2018; 62:18–27. 29413806 10.1016/j.dnarep.2018.01.004 PMC5811311

[2] Smith K.C. Dose dependent decrease in extractability of DNA from bacteria following irradiation with ultraviolet light or with visible light plus dye. Biochem. Biophys. Res. Commun. 1962; 8:157–163. 13914317 10.1016/0006-291x(62)90255-3

[3] Fielden J. , Ruggiano A., Popović M., Ramadan K. DNA protein crosslink proteolysis repair: from yeast to premature ageing and cancer in humans. DNA Repair (Amst.). 2018; 71:198–204. 30170832 10.1016/j.dnarep.2018.08.025 PMC6219452

[4] Hu Q. , Klages-Mundt N., Wang R., Lynn E., Kuma Saha L., Zhang H., Srivastava M., Shen X., Tian Y., Kim H. et al. The ARK assay is a sensitive and versatile method for the global detection of DNA-protein crosslinks. Cell Rep. 2020; 30:1235–1245. 31995761 10.1016/j.celrep.2019.12.067 PMC7069250

[5] Ide H. , Nakano T., Shoulkamy M.I., Salem A.M.H. Formation, repair, and biological effects of DNA–Protein cross-link damage. Advances in DNA Repair. InTech. 2015; 43–80.

[6] Stingele J. , Bellelli R., Boulton S.J. Mechanisms of DNA-protein crosslink repair. Nat. Rev. Mol. Cell Biol. 2017; 18:563–573. 28655905 10.1038/nrm.2017.56

[7] Wei X. , Peng Y., Bryan C., Yang K. Mechanisms of DNA−protein cross-link formation and repair. Biochim. Biophys. Acta. 2021; 1869:140669. 10.1016/j.bbapap.2021.140669 33957291

[8] Stingele J. , Schwarz M.S., Bloemeke N., Wolf P.G., Jentsch S. A DNA-dependent protease involved in DNA-protein crosslink repair. Cell. 2014; 158:327–338. 24998930 10.1016/j.cell.2014.04.053

[9] Lopez-Mosqueda J. , Maddi K., Prgomet S., Kalayil S., Marinovic-Terzic I., Terzic J., Dikic I. SPRTN is a mammalian DNA-binding metalloprotease that resolves DNA-protein crosslinks. Elife. 2016; 5:e21491. 27852435 10.7554/eLife.21491 PMC5127644

[10] Vaz B. , Popovic M., Newman J.A., Fielden J., Aitkenhead H., Halder S., Singh A.N., Vendrell I., Fischer R., Torrecilla I. et al. Metalloprotease SPRTN/DVC1 orchestrates replication-coupled DNA-protein crosslink repair. Mol. Cell. 2016; 64:704–719. 27871366 10.1016/j.molcel.2016.09.032 PMC5128727

[11] Zhang H. , Xiong Y., Chen J. DNA–protein cross-link repair: what do we know now?. Cell. Biosci. 2020; 10:3. 31921408 10.1186/s13578-019-0366-z PMC6945406

[12] Ruggiano A. , Ramadan K. DNA–protein crosslink proteases in genome stability. Commun. Biol. 2021; 4:11. 33398053 10.1038/s42003-020-01539-3 PMC7782752

[13] Ruggiano A. , Ramadan K. The trinity of SPRTN protease regulation. Trends Biochem. Sci. 2021; 46:2–4. 33183910 10.1016/j.tibs.2020.10.007

[14] Fielden J. , Wiseman K., Torrecilla I., Li S., Hume S., Chiang S.-C., Ruggiano A., Narayan Singh A., Freire R., Hassanieh S. et al. TEX264 coordinates p97- and SPRTN-mediated resolution of topoisomerase 1-DNA adducts. Nat. Commun. 2020; 11:1274. 32152270 10.1038/s41467-020-15000-w PMC7062751

[15] Huang J. , Zhou Q., Gao M., Nowsheen S., Zhao F., Kim W., Zhu Q., Kojima Y., Yin P., Zhang Y. et al. Tandem deubiquitination and acetylation of SPRTN promotes DNA-protein crosslink repair and protects against aging. Mol. Cell. 2020; 79:824–835. 32649882 10.1016/j.molcel.2020.06.027 PMC7484104

[16] Zhao S. , Kieser A., Li H.-Y.Y., Reinking H.K., Weickert P., Euteneuer S., Yaneva D., Acampora A.C., Götz M.J., Feederle R. et al. A ubiquitin switch controls autocatalytic inactivation of the DNA–protein crosslink repair protease SPRTN. Nucleic Acids Res. 2021; 49:902–915. 33348378 10.1093/nar/gkaa1224 PMC7826251

[17] Halder S. , Torrecilla I., Burkhalter M.D., Popović M., Fielden J., Vaz B., Oehler J., Pilger D., Lessel D., Wiseman K. et al. SPRTN protease and checkpoint kinase 1 cross-activation loop safeguards DNA replication. Nat. Commun. 2019; 10:3142. 31316063 10.1038/s41467-019-11095-y PMC6637133

[18] Perry M. , Biegert M., Kollala S.S., Mallard H., Su G., Kodavati M., Kreiling N., Holbrook A., Ghosal G. USP11 mediates repair of DNA–protein cross-links by deubiquitinating SPRTN metalloprotease. J. Biol. Chem. 2021; 296:100396. 33567341 10.1016/j.jbc.2021.100396 PMC7960550

[19] Chesner L.N. , Essawy M., Warner C., Campbell C. DNA-protein crosslinks are repaired via homologous recombination in mammalian mitochondria. DNA Repair (Amst.). 2021; 97:103026. 33316746 10.1016/j.dnarep.2020.103026 PMC7855827

[20] Li X. , Heyer W.-D. Homologous recombination in DNA repair and DNA damage tolerance. Cell Res. 2008; 18:99–113. 18166982 10.1038/cr.2008.1 PMC3087377

[21] Nakano T. , Katafuchi A., Matsubara M., Terato H., Tsuboi T., Masuda T., Tatsumoto T., Pack S.P., Makino K., Croteau D.L. et al. Homologous recombination but not nucleotide excision repair plays a pivotal role in tolerance of DNA-protein cross-links in mammalian cells. J. Biol. Chem. 2009; 284:27065–27076. 19674975 10.1074/jbc.M109.019174 PMC2785636

[22] Nakano T. , Morishita S., Katafuchi A., Matsubara M., Horikawa Y., Terato H., Salem A.M.H., Izumi S., Pack S.P., Makino K. et al. Nucleotide excision repair and homologous recombination systems commit differentially to the repair of DNA-protein crosslinks. Mol. Cell. 2007; 28:147–158. 17936711 10.1016/j.molcel.2007.07.029

[23] Reardon J.T. , Cheng Y., Sancar A. Repair of DNA–protein cross-links in mammalian cells. Cell Cycle. 2006; 5:1366–1370. 16775425 10.4161/cc.5.13.2892

[24] Duxin J.P. , Walter J.C. What is the DNA repair defect underlying Fanconi anemia?. Curr. Opin. Cell Biol. 2015; 37:49–60. 26512453 10.1016/j.ceb.2015.09.002 PMC4688103

[25] Blackford A.N. , Jackson S.P ATM, ATR, and DNA-PK: the trinity at the heart of the DNA damage response. Mol. Cell. 2017; 66:801–817. 28622525 10.1016/j.molcel.2017.05.015

[26] Alagoz M. , Chiang S.-C., Sharma A., El-Khamisy S.F. ATM deficiency results in accumulation of DNA-topoisomerase I covalent intermediates in neural cells. PLoS One. 2013; 8:e58239. 23626666 10.1371/journal.pone.0058239 PMC3634035

[27] Nakamura K. , Kustatscher G., Alabert C., Hödl M., Forne I., Völker-Albert M., Satpathy S., Beyer T.E., Mailand N., Choudhary C. et al. Proteome dynamics at broken replication forks reveal a distinct ATM-directed repair response suppressing DNA double-strand break ubiquitination. Mol. Cell. 2021; 81:1084–1099. 33450211 10.1016/j.molcel.2020.12.025 PMC7939521

[28] Tanaka T. , Kurose A., Huang X., Dai W., Darzynkiewicz Z. ATM activation and histone H2AX phosphorylation as indicators of DNA damage by DNA topoisomerase I inhibitor topotecan and during apoptosis. Cell Prolif. 2006; 39:49–60. 16426422 10.1111/j.1365-2184.2006.00364.x PMC6496121

[29] Cristini A. , Park J.-H., Capranico G., Legube G., Favre G., Sordet O. DNA-PK triggers histone ubiquitination and signaling in response to DNA double-strand breaks produced during the repair of transcription-blocking topoisomerase I lesions. Nucleic Acids Res. 2016; 44:1161–1178. 26578593 10.1093/nar/gkv1196 PMC4756817

[30] Maskey R.S. , Flatten K.S., Sieben C.J., Peterson K.L., Baker D.J., Nam H.J., Kim M.S., Smyrk T.C., Kojima Y., Machida Y. et al. Spartan deficiency causes accumulation of topoisomerase 1 cleavage complexes and tumorigenesis. Nucleic Acids Res. 2017; 45:4564–4576. 28199696 10.1093/nar/gkx107 PMC5416836

[31] Kiianitsa K. , Maizels N. A rapid and sensitive assay for DNA–protein covalent complexes in living cells. Nucleic Acids Res. 2013; 41:e104. 23519618 10.1093/nar/gkt171 PMC3643584

[32] Barker S. , Murray D., Zheng J., Li L., Weinfeld M. A method for the isolation of covalent DNA–protein crosslinks suitable for proteomics analysis. Anal. Biochem. 2005; 344:204–215. 16091282 10.1016/j.ab.2005.06.039

[33] Smith P.K. , Krohn R.I., Hermanson G.T., Mallia A.K., Gartner F.H., Provenzano M.D., Fujimoto E.K., Goeke N.M., Olson B.J., Klenk D.C. Measurementof protein using bicinchoninic acid. Anal. Biochem. 1985; 150:76–85. 3843705 10.1016/0003-2697(85)90442-7

[34] Borchert N. , Dieterich C., Krug K., Schütz W., Jung S., Nordheim A., Sommer R.J., Macek B. Proteogenomics of Pristionchus pacificus reveals distinct proteome structure of nematode models. Genome Res. 2010; 20:837–846. 20237107 10.1101/gr.103119.109 PMC2877580

[35] Rappsilber J. , Mann M., Ishihama Y. Protocol for micro-purification, enrichment, pre-fractionation and storage of peptides for proteomics using StageTips. Nat. Protoc. 2007; 2:1896–1906. 17703201 10.1038/nprot.2007.261

[36] Schmitt M. , Sinnberg T., Nalpas N.C., Maass A., Schittek B., Macek B. Quantitative proteomics links the intermediate filament nestin to resistance to targeted BRAF inhibition in melanoma cells. Mol. Cell. Proteomics. 2019; 18:1096–1109. 30890564 10.1074/mcp.RA119.001302 PMC6553926

[37] Cox J. , Mann M. MaxQuant enables high peptide identification rates, individualized p.P.B.-range mass accuracies and proteome-wide protein quantification. Nat. Biotechnol. 2008; 26:1367–1372. 19029910 10.1038/nbt.1511

[38] Cox J. , Neuhauser N., Michalski A., Scheltema R.A., Olsen J.V., Mann M. Andromeda: a peptide search engine integrated into the MaxQuant environment. J. Proteome Res. 2011; 10:1794–1805. 21254760 10.1021/pr101065j

[39] R Core Team R: a language and environment for statistical computing. R Foundation for Statistical Computing. 2020; Vienna, Austria.

[40] Mi H. , Ebert D., Muruganujan A., Mills C., Albou L.-P., Mushayamaha T., Thomas P.D. PANTHER version 16: a revised family classification, tree-based classification tool, enhancer regions and extensive API. Nucleic. Acids. Res. 2021; 49:D394–D403. 33290554 10.1093/nar/gkaa1106 PMC7778891

[41] Larsson J. , Godfrey A.J.R., Gustafsson P., Eberly D.H., Huber E., Slowikowski K., Privé F. A Case Study in Fitting Area-Proportional Euler Diagrams with Ellipses Using eulerr. Proceedings of International Workshop on Set Visualization and Reasoning. 2018; 2116:84–91.

[42] Perez-Riverol Y. , Bai J., Bandla C., García-Seisdedos D., Hewapathirana S., Kamatchinathan S., Kundu D.J., Prakash A., Frericks-Zipper A., Eisenacher M. et al. The PRIDE database resources in 2022: a hub for mass spectrometry-based proteomics evidences. Nucleic Acids Res. 2022; 50:D543–D552. 34723319 10.1093/nar/gkab1038 PMC8728295

[43] Schneider C.A. , Rasband W.S., Eliceiri K.W. NIH image to ImageJ: 25 years of image analysis. Nat. Methods. 2012; 9:671–675. 22930834 10.1038/nmeth.2089 PMC5554542

[44] Gyori B.M. , Venkatachalam G., Thiagarajan P.S., Hsu D., Clement M.-V. OpenComet: an automated tool for comet assay image analysis. Redox. Biol. 2014; 2:457–465. 24624335 10.1016/j.redox.2013.12.020 PMC3949099

[45] Møller P. , Azqueta A., Boutet-Robinet E., Koppen G., Bonassi S., Milić M., Gajski G., Costa S., Teixeira J.P., Costa Pereira C. et al. Minimum information for reporting on the Comet assay (MIRCA): recommendations for describing comet assay procedures and results. Nat. Protoc. 2020; 15:3817–3826. 33106678 10.1038/s41596-020-0398-1 PMC7688437

[46] Pachva M.C. , Kisselev A.F., Matkarimov B.T., Saparbaev M., Groisman R. DNA-histone cross-links: formation and repair. Front. Cell Dev. Biol. 2020; 8:607045. 33409281 10.3389/fcell.2020.607045 PMC7779557

[47] Mórocz M. , Zsigmond E., Tóth R., Enyedi M.Z., Pintér L., Haracska L. DNA-dependent protease activity of human Spartan facilitates replication of DNA–protein crosslink-containing DNA. Nucleic. Acids. Res. 2017; 45:3172–3188. 28053116 10.1093/nar/gkw1315 PMC5389635

[48] Pagano M. , Pepperkok R., Verde F., Ansorge W., Draetta G. Cyclin A is required at two points in the human cell cycle. EMBO J. 1992; 11:961–971. 1312467 10.1002/j.1460-2075.1992.tb05135.x PMC556537

[49] Baldin V. , Lukas J., Marcote M.J., Pagano M., Draetta G. Cyclin D1 is a nuclear protein required for cell cycle progression in G1. Genes Dev. 1993; 7:812–821. 8491378 10.1101/gad.7.5.812

[50] Hans F. , Dimitrov S. Histone H3 phosphorylation and cell division. Oncogene. 2001; 20:3021–3027. 11420717 10.1038/sj.onc.1204326

[51] Sun Y. , Chen J., Huang S.N., Su Y.P., Wang W., Agama K., Saha S., Jenkins L.M., Pascal J.M., Pommier Y. PARylation prevents the proteasomal degradation of topoisomerase I DNA-protein crosslinks and induces their deubiquitylation. Nat. Commun. 2021; 12:5010. 34408146 10.1038/s41467-021-25252-9 PMC8373905

[52] Zhitkovich A. , Costa M. A simple, sensitive assay to detect DNA–protein cromlinks in intact cells and in vivo. Carcinogenesis. 1992; 13:1485–1489. 1499101 10.1093/carcin/13.8.1485

[53] Larsen N.B. , Gao A.O., Sparks J.L., Gallina I., Wu R.A., Mann M., Räschle M., Walter J.C., Duxin J.P. Replication-coupled DNA-protein crosslink repair by SPRTN and the proteasome in Xenopus egg extracts. Mol. Cell. 2019; 73:574–588. 30595436 10.1016/j.molcel.2018.11.024 PMC6375733

[54] Cleaver J.E. , Feeney L., Revet I. Phosphorylated H2Ax is not an unambiguous marker for DNA double-strand breaks. Cell Cycle. 2011; 10:3223–3224. 21921674 10.4161/cc.10.19.17448

[55] An J. , Huang Y.-C., Xu Q.-Z., Zhou L.-J., Shang Z.-F., Huang B., Wang Y., Liu X.-D., Wu D.-C., Zhou P.-K. DNA-pkcs plays a dominant role in the regulation of H2AX phosphorylation in response to DNA damage and cell cycle progression. BMC Mol. Biol. 2010; 11:18. 20205745 10.1186/1471-2199-11-18 PMC2844398

[56] Podhorecka M. , Skladanowski A., Bozko P. H2AX Phosphorylation: its role in DNA damage response and cancer therapy. J. Nucleic Acids. 2010; 2010:920161. 20811597 10.4061/2010/920161 PMC2929501

[57] Meador J.A. , Zhao M., Su Y., Narayan G., Geard C.R., Balajee A.S. Histone H2AX is a critical factor for cellular protection against DNA alkylating agents. Oncogene. 2008; 27:5662–5671. 18542054 10.1038/onc.2008.187

[58] Marti T.M. , Hefner E., Feeney L., Natale V., Cleaver J.E. H2AX phosphorylation within the G1 phase after UV irradiation depends on nucleotide excision repair and not DNA double-strand breaks. Proc. Natl. Acad. Sci. U.S.A. 2006; 103:9891–9896. 16788066 10.1073/pnas.0603779103 PMC1502549

[59] Stope M. Phosphorylation of histone H2A.X as a DNA-associated biomarker (review). World Acad. Sci. J. 2021; 3:31.

[60] Mao P. , Wyrick J.J. Emerging roles for histone modifications in DNA excision repair. FEMS Yeast Res. 2016; 16:fow090. 27737893 10.1093/femsyr/fow090 PMC5976035

[61] Menolfi D. , Zha S. ATM, ATR and DNA-pkcs kinases—the lessons from the mouse models: inhibition ≠ deletion. Cell Biosci. 2020; 10:8. 32015826 10.1186/s13578-020-0376-x PMC6990542

[62] Yue X. , Bai C., Xie D., Ma T., Zhou P.-K. DNA-pkcs: a multi-faceted player in DNA damage response. Front Genet. 2020; 11:607428. 33424929 10.3389/fgene.2020.607428 PMC7786053

[63] Kinner A. , Wu W., Staudt C., Iliakis G. Gamma-H2AX in recognition and signaling of DNA double-strand breaks in the context of chromatin. Nucleic Acids Res. 2008; 36:5678–5694. 18772227 10.1093/nar/gkn550 PMC2553572

[64] Bewersdorf J. , Bennett B.T., Knight K.L. H2AX chromatin structures and their response to DNA damage revealed by 4Pi microscopy. Proc. Natl. Acad. Sci. U.S.A. 2006; 103:18137–18142. 17110439 10.1073/pnas.0608709103 PMC1636994

