# Supplementary material for: SPRTN-dependent DPC degradation precedes repair of damaged DNA: a proof of concept revealed by the STAR assay
Source: Nucleic Acids Res. 2023 Jan 31;51(6):e35. doi: 10.1093/nar/gkad022 (PMC10085693; doi:10.1093/nar/gkad022)
Supplement: gkad022_Supplemental_Files [file gkad022_supplemental_files.zip › Antibody list.docx]

**The list of antibodies**

| Antibody | Company | Identifier |
| --- | --- | --- |
| H3 | Santa Cruz Biotechnology | sc-517576 |
| NDHII (DDX9) | Santa Cruz Biotechnology | sc-137232 |
| β-Actin (ACTN) | Sigma-Aldrich | A5316-2ML |
| GAPDH | Proteintech | 10494-1-AP |
| p68 RNA helicase (DDX5) | Santa Cruz Biotechnology | scc-365164 |
| Vinkulin | Sigma-Aldrich | V9264 |
| HSP90 | Santa Cruz Biotechnology | sc-69703 |
| α-Tubulin (TUBA) | Sigma-Aldrich | T9026-2ML |
| VCP | Novus Biologicals | NB100-1557 |
| p73 | Cell Signaling Technology | #14620S |
| HDAC1 | Santa Cruz Biotechnology | sc-7872 |
| PARP1 | Cell Signaling Technology | #9542S |
| GRP78 | Proteintech | 11587-1-AP |
| PKM | Cusabio | CSB-PA018072DA01HU |
| XRCC3 | Proteintech | 18494-1-AP |
| TOPO1 | DSHB | CPTC-TOP1-1-S |
| yH2Ax | Cell Signaling Technology | #9718S |
| ACTN4 | Cusabio | CSB-PA00814A0Rb |
