# Supplementary material for: SPRTN-dependent DPC degradation precedes repair of damaged DNA: a proof of concept revealed by the STAR assay
Source: Nucleic Acids Res. 2023 Jan 31;51(6):e35. doi: 10.1093/nar/gkad022 (PMC10085693; doi:10.1093/nar/gkad022)
Supplement: gkad022_Supplemental_Files [file gkad022_supplemental_files.zip › Supplementary figures and tables.pdf]

Supplementary material

SPRTN-dependent DPC degradation precedes repair of damaged DNA: A proof of concept revealed by the STAR assay

Authors: Mateo Glumac, Mirjana Polović, Anja Batel, Andrea Gelemanović, Boris Maček, Ana Velić, Ivana Marinović-Terzić

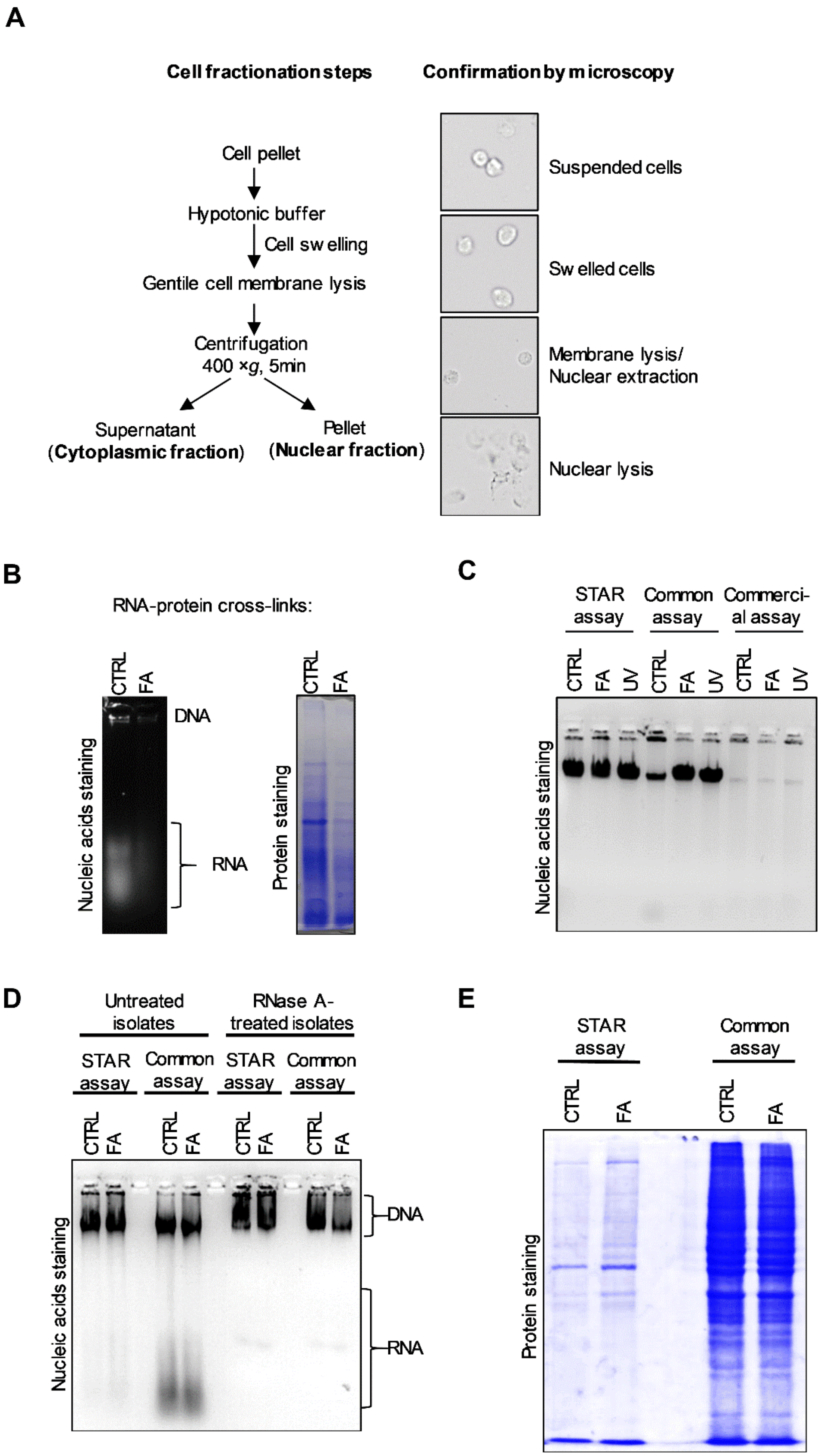
A
Cell fractionation steps
Confirmation by microscopy
Cell pellet
Hypotonic buffer
Cell swelling
Gentile cell membrane lysis
Centrifugation
400 ×g, 5min
Supernatant
(Cytoplasmic fraction)
Pellet
(Nuclear fraction)
Suspended cells
Swelled cells
Membrane lysis/
Nuclear extraction
Nuclear lysis
B
RNA-protein cross-links:
CTRL
FA
DNA
RNA
Nucleic acids staining
Protein staining
C
STAR assay
Common assay
Commerci-al assay
CTRL
FA
UV
Nucleic acids staining
D
Untreated isolates
RNase A-treated isolates
STAR assay
Common assay
CTRL
FA
DNA
RNA
Nucleic acids staining
E
STAR assay
Common assay
CTRL
FA
Protein staining

Figure S1: RNA-protein crosslinks are the major source of protein background signal.

(A) Scheme and accompanying microscopy photographs for cell fractionation protocol used in this study. (B) Agarose gel electrophoresis of RNA obtained from buffer 1 by ethanol precipitation (left). RNA was cleaned with buffer 2 following the STAR assay protocol and analyzed by ethidium bromide staining. Protein content was determined by SDS-PAGE and CBB staining of the same RNA isolates (right). (C) Isolates from Figure 2A after RNase treatment demonstrate the difference in the amount of RNA contamination in isolates from compared isolation protocols. (D) Agarose gel electrophoresis and ethidium bromide staining of DPCs isolated from FA-treated U2OS cells. DPCs were isolated by the STAR and the common assays. RNase was used to demonstrate the presence of RNA in the samples. (E) SDS-PAGE and CBB staining of the same DPC isolates from (C).

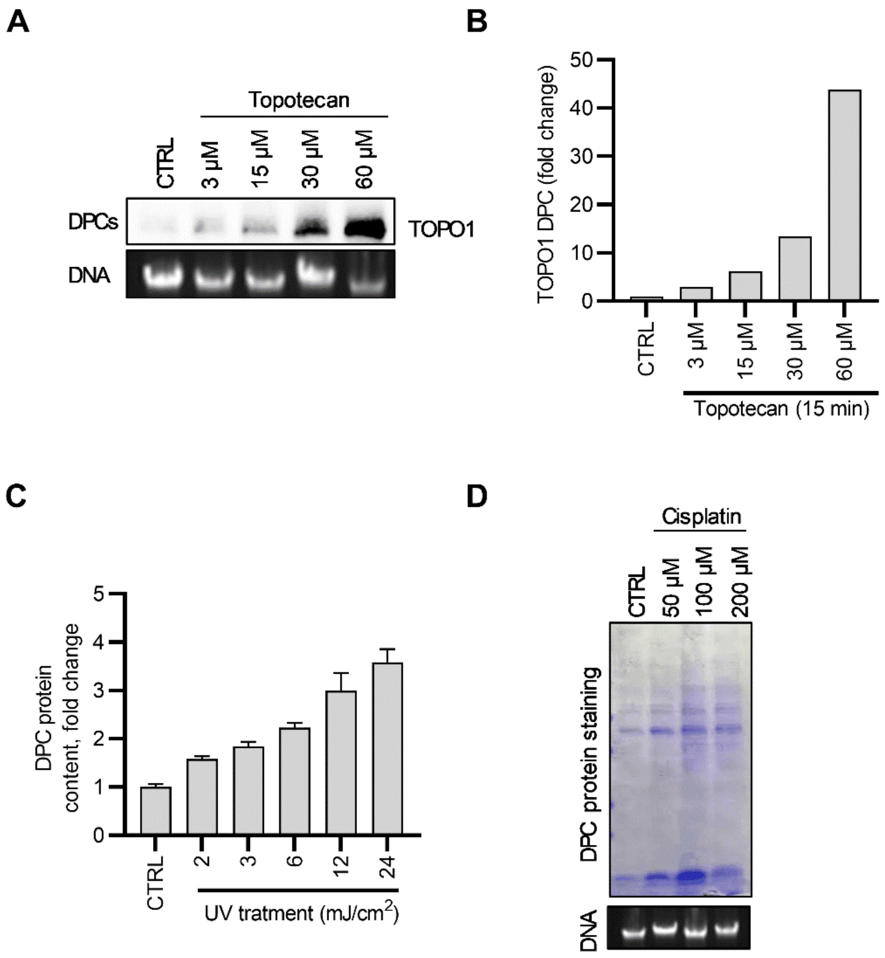

Figure S2: The STAR assay isolation of DPCs induced by TOP, UV, and cisplatin.

DPC formation was induced by TOP treatment for 15 minutes in indicated concentrations and compared to untreated control cells. The effect of TOP treatment was measured as (A) TOP-induced Topoisomerase 1 trapped onto DNA molecule and probed by immunoblotting, using DNA as a loading control; and (B) the fold increase in topoisomerase 1 positive DPCs. (C) UV-induced DPCs were isolated by the STAR assay and quantified using the BCA assay. (D) Cisplatin-induced DPCs were isolated by the STAR assay and evaluated on SDS-PAGE CBB stained gel.

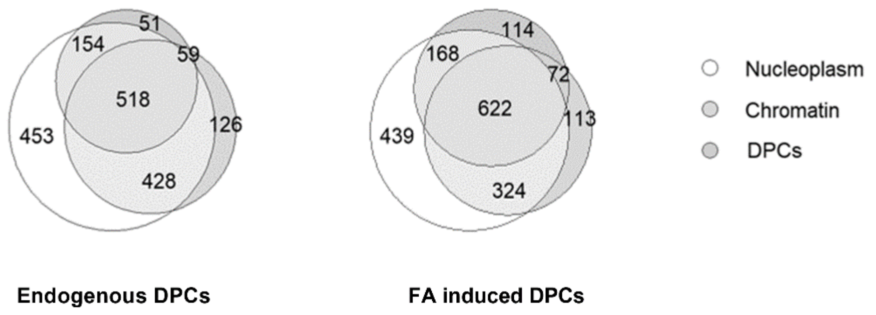

Figure S3: The MS analysis of proteins identified in nucleoplasm or chromatin fractions and DPC isolates.

Venn diagram presentation of qualitative composition of endogenous and FA-induced DPCs analyzed by MS, showing the overlap between proteins detected in DPCs, and proteins detected in nucleoplasmic and chromatin nuclear fractions.

A

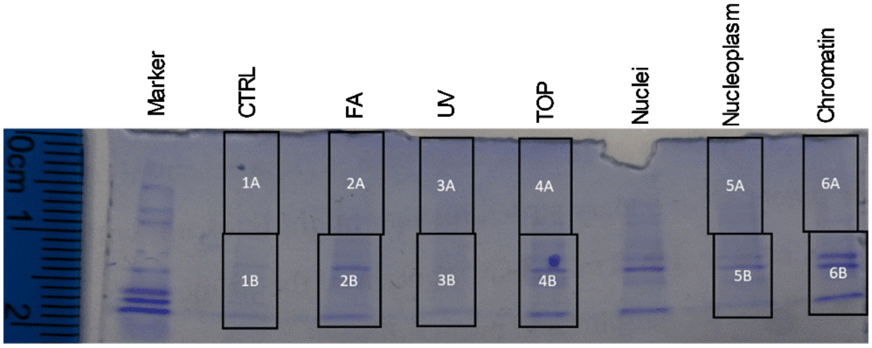
Marker
CTRL
FA
UV
TOP
Nuclei
Nucleoplasm
Chromatin
0cm
1
2
1A
2A
3A
4A
5A
6A
1B
2B
3B
4B
5B
6B

B

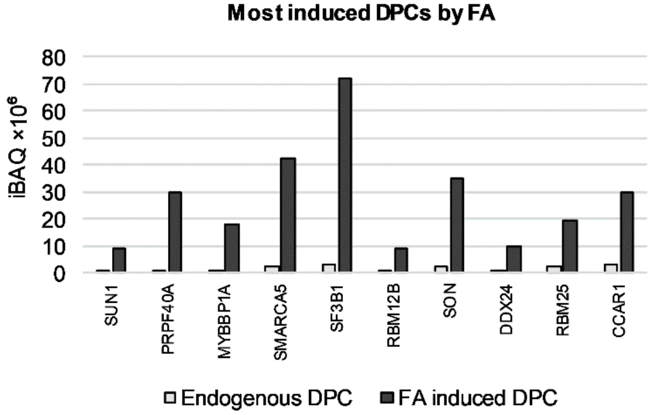
Most induced DPCs by FA
iBAQ ×10^6
0
10
20
30
40
50
60
70
80
SUN1
PRPF40A
MYBBP1A
SMARCA5
SF3B1
RBM12B
SON
DDX24
RBM25
CCAR1
Endogenous DPC
FA induced DPC

C

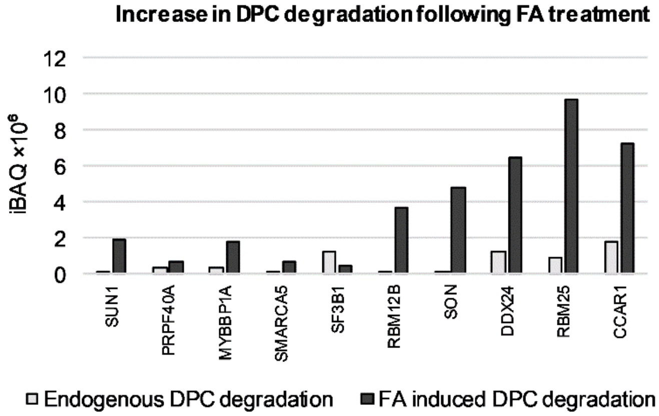
Increase in DPC degradation following FA treatment
iBAQ ×10^6
0
2
4
6
8
10
12
SUN1
PRPF40A
MYBBP1A
SMARCA5
SF3B1
RBM12B
SON
DDX24
RBM25
CCAR1
Endogenous DPC degradation
FA induced DPC degradation

Figure S4: Identification of proteolytically created DNA-peptide crosslinks by MS.

(A) Preparation of proteins for MS analysis by SDS-PAGE and CBB staining. Each lane was cut into two sections at 60 kDa size (determined by protein marker). Sections were analyzed separately. (B) Top 10 most induced DPCs after FA treatment (400 μM, 15 min). The data presented is the sum of iBAQ obtained in the upper and bottom gel sections. (C) Top 10 most proteolytically degraded DPCs after FA treatment (400 μM, 15 min). The data presented is the proportion of detected peptides specific for each protein found in the lower gel section.

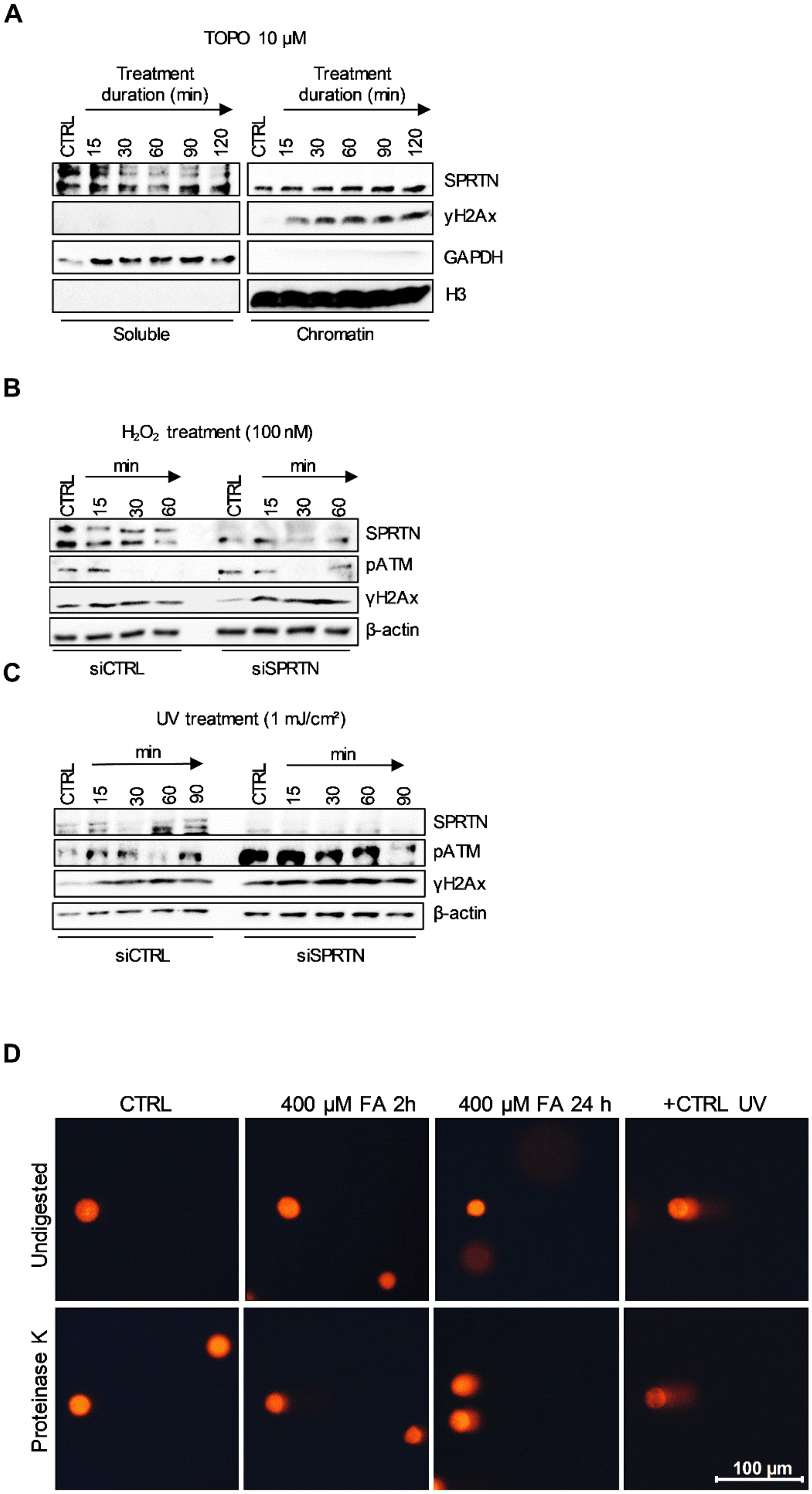

A
TOPO 10 μM
Treatment duration (min)
Treatment duration (min)
CTRL
15
30
60
90
120
CTRL
15
30
60
90
120
SPRTN
γH2Ax
GAPDH
H3
Soluble
Chromatin
B
H2O2 treatment (100 nM)
min
min
CTRL
15
30
60
CTRL
15
30
60
SPRTN
pATM
γH2Ax
β-actin
siCTRL
siSPRTN
C
UV treatment (1 mJ/cm²)
min
min
CTRL
15
30
60
90
CTRL
15
30
60
90
SPRTN
pATM
γH2Ax
β-actin
siCTRL
siSPRTN
D
CTRL
400 μM FA 2h
400 μM FA 24 h
+CTRL UV
Undigested
Proteinase K
100 μm

Figure S5: γH2Ax activation is not dependent on SPRTN in the absence of DPCs

(A) DNA damage signaling and SPRTN expression were analyzed by WB in soluble and chromatin fractions obtained from cells treated with 10 μM of TOP for the indicated times. GAPDH and H3 were used as a loading control for appropriate fractions. (B) The influence of SPRTN on activation of pATM and γH2Ax induced by H_2O_2 treatment (100 nM). SPRTN expression was reduced by siRNA. Actin was used as a loading control. (C) The influence of SPRTN on activation of pATM and γH2Ax induced by UV treatment (1 mJ/cm^2). SPRTN expression was reduced by siRNA. Actin was used as a loading control. (D) Optimization of Comet assay for evaluating DPC-induced DNA damage. Proof of concept for Proteinase K digestion.

Supplementary table

Table S1: The analysis of formaldehyde-induced DPC formation and repair in control cells and cells with silenced SPRTN.

	DPC formation	DPC repair			
Comparison / Sample	0 min vs Control	30 min vs 0 min	60 min vs 0 min	90 min vs 0 min	120 min vs 0 min
si-CTRL	P ≤ 0.01	N.S.	N.S.	P ≤ 0.05	P ≤ 0.001
si-SPRTN	P ≤ 0.01	N.S.	N.S.	N.S.	P ≤ 0.05

T-test for independent samples.
